# BMP-dependent synaptic development requires Abi-Abl-Rac signaling of BMP receptor macropinocytosis

**DOI:** 10.1038/s41467-019-08533-2

**Published:** 2019-02-08

**Authors:** Najin Kim, Sungdae Kim, Minyeop Nahm, Danielle Kopke, Joohyung Kim, Eunsang Cho, Min-Jung Lee, Mihye Lee, Seung Hyun Kim, Kendal Broadie, Seungbok Lee

**Affiliations:** 1 Interdisciplinary Graduate Program in Genetic Engineering, Seoul National University, Seoul, 08826 Korea; 2 Department of Neurology, Hanyang University College of Medicine, Seoul, 04763 Korea; 3 Departments of Biological Sciences, Cell and Developmental Biology, and Pharmacology, Vanderbilt Brain Institute, Vanderbilt Kennedy Center for Research on Human Development, Vanderbilt University, Nashville, TN 37232 USA; 4 Department of Brain and Cognitive Sciences, Seoul National University, Seoul, 08826 Korea; 5 Department of Cell and Developmental Biology and Dental Research Institute, Seoul National University, Seoul, 08826 Korea

## Abstract

Retrograde BMP *trans*-synaptic signaling is essential for synaptic development. Despite the importance of endocytosis-regulated BMP receptor (BMPR) control of this developmental signaling, the mechanism remains unknown. Here, we provide evidence that Abelson interactor (Abi), a substrate for Abl kinase and component of the SCAR/WAVE complex, links Abl and Rac1 GTPase signaling to BMPR macropinocytosis to restrain BMP-mediated synaptic development. We find that Abi acts downstream of Abl and Rac1, and that BMP ligand Glass bottom boat (Gbb) induces macropinocytosis dependent on Rac1/SCAR signaling, Abl-mediated Abi phosphorylation, and BMPR activation. Macropinocytosis acts as the major internalization route for BMPRs at the synapse in a process driven by Gbb activation and resulting in receptor degradation. Key regulators of macropinocytosis (Rabankyrin and CtBP) control BMPR trafficking to limit BMP *trans*-synaptic signaling. We conclude that BMP-induced macropinocytosis acts as a BMPR homeostatic mechanism to regulate BMP-mediated synaptic development.

## Abstract (web summary)

BMP ligands act as retrograde signalling molecules to regulate presynaptic development, and regulation of BMP receptors by endocytosis may be an important component of this signalling pathway. Here, the authors show that Abi-mediated macropinocytosis of BMP receptors in Drosophila larva and contributes to neuromuscular development.

## Introduction

Bidirectional communication between presynaptic and postsynaptic compartments mediated by *trans*-synaptic signals is fundamental to both development and plasticity of neuronal synapses^1,2^. Bone morphogenetic protein (BMP) ligands are key retrograde signals (post to pre) that regulate presynaptic development in *Drosophila* and mammals^3,4^. At the *Drosophila* neuromuscular junction (NMJ), muscle-derived BMP Glass bottom boat (Gbb)^5^ activates a presynaptic receptor complex composed of type II BMP receptor (BMPR) Wishful thinking (Wit) and either type I BMPR Thickveins (Tkv) or Saxophone (Sax)^6–8^. BMPR activation causes phosphorylation of the R-Smad Mothers against decapentaplegic (Mad), and phosphorylated Mad (P-Mad) together with co-Smad Medea (Med) enters the nucleus to regulate transcription^9^. Key targets of this BMP pathway include *trio*^10^, encoding a Rac1 guanine-nucleotide exchange factor, and *Drosophila fragile X mental retardation 1* (*dFmr1*)^11^, encoding a negative regulator of Futsch/MAP1B translation^12^. Mutations disrupting Gbb signaling cause synaptic undergrowth^5–8,10,11^, whereas elevated Gbb signaling results in NMJ overgrowth characterized by satellite bouton formation and hyperbudding^11,13^. Thus BMP signaling has an instructive role in presynaptic development

Increasing evidence suggests that presynaptic BMP signaling is regulated by endocytosis and trafficking of BMPRs. Mutations in endocytic regulators, including *Dynamin* (*shibire*), *Endophilin*, and *Dap160*, increase P-Mad levels with NMJ overgrowth and excessive satellite bouton formation^13,14^. Similarly, synaptic development is upregulated by loss of Spichthyin and Spartin proteins involved in endosomal sorting of BMPRs for lysosomal degradation^11,15^. However, the endocytic pathway mediating BMPR internalization/downregulation is uncharacterized. A candidate is macropinocytosis initiated by actin-driven plasma membrane ruffles, which subsequently develop into large (0.2–5 μm) endocytic macropinosome vesicles^16^. In mammalian cells, membrane ruffling and macropinosome formation are dependent on Rac1 GTPase and the SCAR/WAVE complex^17,18^, which relays Rac1 signaling to actin nucleator Arp2/3 (refs. ^19,20^). SCAR proteins form a multimeric complex comprising Nap1/Kette, Sra-1/CYFIP, HSPC300, and Abelson (Abl) interacting protein (Abi)^21–23^, which acts as a scaffold between SCAR and Kette^22,24,25^. The binding of Rac1 to Sra-1/CYFIP stimulates SCAR activity toward the Arp2/3 complex^26^. However, little is known about SCAR complex targeting to macropinosomes to control actin dynamics, and nothing is known about macropinocytosis in synaptic development.

Abi is phosphorylated by the non-receptor tyrosine kinase Abl, which is essential for membrane localization, protein stability, and lamella formation activity^27–31^. Interestingly, loss of Abl or SCAR complex components causes satellite bouton formation and hyperbudding at the *Drosophila* NMJ^32–37^. This suggests a mechanistic link between Abl/SCAR signaling and BMP-dependent synaptic growth. In testing this hypothesis, we show here that Abi acts downstream of Rac1 to regulate synaptic development through the SCAR complex. We also show that this Abi role absolutely depends on phosphorylation mediated by Abl. Our genetic data suggest Abl-Abi and Rac1-SCAR signaling restrain synaptic growth via inhibition of presynaptic BMP signaling. Importantly, we show that Gbb induces synaptic macropinocytosis in a BMPR-dependent mechanism, with induction impaired by disrupting both Abl-Abi and Rac1-SCAR pathways. Moreover, we demonstrate that macropinocytosis is the predominant internalization route for BMPRs in the presence of Gbb ligand and indispensable for efficient BMPR degradation. Finally, we discover that two known regulators of macropinocytosis, Rabankyrin and CtBP, are required for normal BMP signaling in synaptic development. Together, these findings establish an unexpected role for Gbb-induced macropinocytosis in the downregulation of synaptic BMPRs.

## Results

### Abi has essential functions in the neuromusculature

**Fig. 1:**
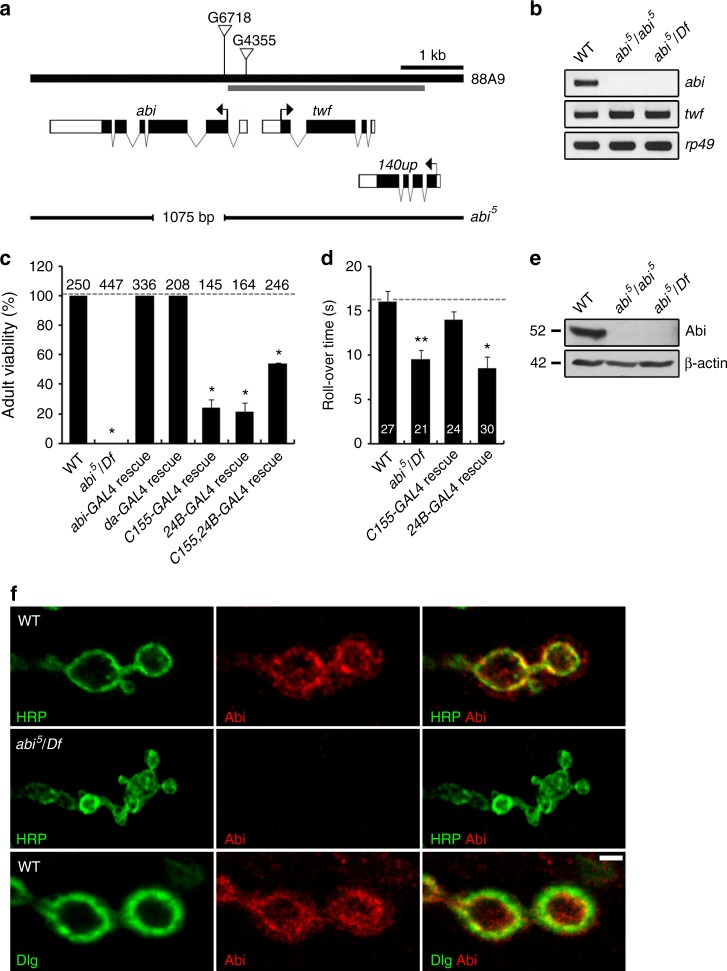
Characterization of *Drosophila abi* gene, mutants, and neuromuscular junction (NMJ) expression. **a** Genomic organization of the *Drosophila abi* locus showing exon/intron organization of *abi* and two neighboring genes (*twf* and *140up*), position of P-elements (G6718 and G4355), and *abi*^*5*^ deletion generated by G6718 excision. Untranslated regions, white boxes; translated regions, black boxes; translation start sites, arrows. Gray bar represents the *abi*-*GAL4* promoter region. **b** Reverse transcription-PCR analysis of *abi*, *twf*, and *rp49* RNA expression in wild type (WT; *w*^*1118*^), *abi*^*5*^, and *abi*^*5*^/*Df*. **c** Quantification of adult viability for wild type, *abi*^*5*^/*Df*, *abi*-*GAL4*/+; *UAS*-*HA*-*abi*,*Df*/*abi*^*5*^ (*abi*-*GAL4* rescue), *da*-*GAL4*,*abi*^*5*^/*UAS*-*HA*-*abi*,*Df* (*da*-*GAL4* rescue), *C155*-*GAL4*/+; *UAS*-*HA*-*abi*,*Df*/*abi*^*5*^ (*C155*-*GAL4* rescue), *24B*-*GAL4*,*abi*^*5*^/*UAS*-*HA*-*abi*,*Df* (*24B*-*GAL4* rescue), and *C155*-*GAL4*/+; *24B*-*GAL4*,*abi*^*5*^/*UAS*-*HA*-*abi*,*Df* (*C155*, *24B*-*GAL4* rescue) animals. The number of *abi*^*5*^/*Df* flies is given as a percentage of the expected viability, which is half the number of adults carrying a balancer chromosome. Values are from three independent experiments and presented as percentages of wild type. **d** Quantification of response time in the larval roll-over assay for the indicated genotypes. **e** Western blot of third instar larval extracts probed with anti-Abi and anti-β-actin. Numbers are molecular masses in kDa. **f** Abi is enriched at NMJ boutons. Single confocal slices of NMJ 6/7 in wild type and *abi*^*5*^/*Df* co-labeled for anti-Abi and anti-HRP (top) or anti-Dlg (bottom). Scale bars: 2 μm. Bar graphs show mean ± s.e.m. The number of animals analyzed in at least three experiments is indicated above (**c**) or inside (**d**) bars. Statistical analyses were performed by one-way analysis of variance with Tukey–Kramer post hoc test. Comparisons are with wild type (*P < 0.001; **P < 0.01)

In a genetic screen for mutations affecting synaptic growth and architecture of the *Drosophila* NMJ^38^, we identified two EP insertions (G6718, G4355) in the *abi* gene (Fig. 1a). Third instar larvae homozygous for each insertion display more extensive NMJ architecture than the genetic control (*w*^*1118*^). To generate *abi* null alleles, we excised the G6718 transposon and isolated the imprecise excision *abi*^*5*^ (1075-bp deletion), which removes large portions of the second and third exons (Fig. 1a). Expression of the *abi* transcript is abolished in homozygous *abi*^*5*^ mutants or in animals heterozygous with an *abi* deficiency (*Df(3R)su(Hw)7*; hereafter *Df*), while the neighboring *twinfilin* (*twf*) gene is normally expressed (Fig. 1b). The mutants show pupal lethality, consistent with previously established *abi* requirements^33,39^. Expression of *UAS*-*HA*-*abi* under control of an *abi* promoter–*GAL4* (*abi*-*GAL4*) or ubiquitous *daughterless* (*da*)–*GAL4* driver completely rescues the lethality of *abi*^*5*^/*Df* mutants (Fig. 1c). Importantly, expression of *UAS*-*HA*-*abi* using the combined pan-neuronal *C155*-*GAL4* and muscular *24B*-*GAL4* drivers very significantly restores *abi* null viability, while expression using each GAL4 alone results in weaker rescue (Fig. 1c), indicating that Abi has essential functions in the neuromusculature. The *abi* mutants exhibit impaired coordinated motor behavior in the roll-over assay. In this assay, we measured the time that individual third instar larvae take to right from a totally inverted position (ventral up) to the normal position (ventral down)^40^. *abi*^*5*^/*Df* larvae show faster roll-over than wild-type controls (*w*^*1118*^: 15.9 ± 2.4 s; *abi*^*5*^/*Df*: 9.5 ± 1.0 s; mean ± s.e.m., *P* < 0.01; Fig. 1d). This behavioral phenotype is fully rescued by *UAS*-*HA*-*abi* expression under control of *C155*-*GAL4* (13.9 ± 1.2 s; *P* = 0.63 from wild type; Fig. 1d), but not of *24B*-*GAL4* (8.5 ± 1.6 s; *P* < 0.001 from wild type; Fig. 1d), strongly supporting a neuronal role for Abi.

### Abi localizes to the sub-membrane cortex within NMJ boutons

Abi has been localized to several axonal tracts in developing embryonic and larval central nervous systems^33^. To investigate Abi expression at NMJ synapses, we generated a new specific antibody. Immunoblots of wild-type third instar larval lysates detect a 52 kDa band, which is completely absent from *abi*^*5*^/*abi*^*5*^ and *abi*^*5*^/*Df* mutants (Fig. 1e). Anti-Abi labeling reveals strong expression at all types of larval NMJ terminals (types I–III). Expression is not uniformly distributed but rather localized to punctate domains associated with the horseradish peroxidase (HRP)-labeled presynaptic membrane and internal cortical regions within boutons (Fig. 1f). A low number of Abi punctae appear postsynaptic, outside the HRP-labeled presynaptic membrane. Null *abi* NMJs display no labeling with anti-Abi, demonstrating the antibody specificity (Fig. 1f, middle). Consistent with the primarily presynaptic expression pattern, postsynaptic subsynaptic reticulum (SSR) labeling with an antibody to the Discs large (Dlg) scaffold largely surrounds the Abi expression domain (Fig. 1f, bottom). Thus Abi is primarily localized beneath the presynaptic membrane at NMJ boutons.

### Abi is required for normal synaptic structure and function

**Fig. 2:**
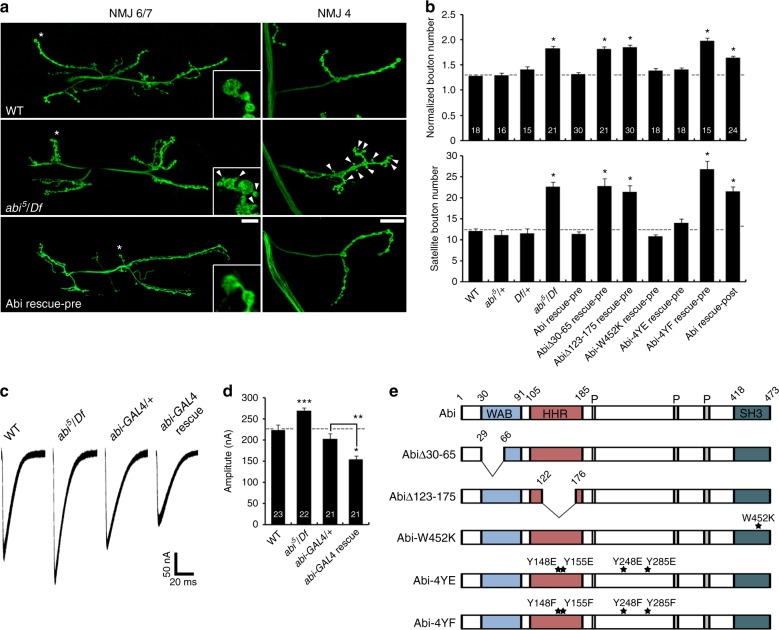
*abi* controls synaptic structure and function at the neuromuscular junction (NMJ). **a** Loss of Abi causes synaptic overgrowth with excessive satellite bouton formation. Sample confocal images of anti-HRP labeled NMJs 6/7 and 4 in wild type, *abi*^*5*^/*Df*, and *C155*-*GAL4*/+; *UAS*-*HA*-*abi*,*Df*/*abi*^*5*^ (Abi rescue-pre). Insets (left panels) show higher magnification views of boutons marked by white asterisks. Satellite boutons are indicated by arrowheads. Scale bars: 20 μm. **b** Quantification of total bouton number normalized to muscle surface area ([#/μm^2^] × 10^3^; top) and satellite bouton number (bottom) at NMJ 6/7 in wild type, *abi*^*5*^/+, *Df*/+, *abi*^*5*^/*Df*, *C155*-*GAL4*/+; *UAS*-*HA*-*abi*,*Df*/*abi*^*5*^ (Abi rescue-pre), *C155*-*GAL4*/+; *UAS*-*HA*-*abi*^*Δ30–65*^/+; *abi*^*5*^/*Df* (AbiΔ30–65 rescue-pre), *C155*-*GAL4*/+; *UAS*-*HA*-*abi*^*Δ123–175*^,*Df*/*abi*^*5*^ (AbiΔ123–175 rescue-pre), *C155*-*GAL4*/+; *UAS*-*HA*-*abi*^*W452K*^,*Df*/*abi*^*5*^ (Abi-W452K rescue-pre), *C155*-*GAL4*/+; *UAS*-*HA*-*abi*^*4YE*^,*Df*/*abi*^*5*^ (Abi-4YE rescue-pre), *C155*-*GAL4*/+; *UAS*-*HA*-*abi*^*4YF*^,*Df*/*abi*^*5*^ (Abi-4YF rescue-pre), *24B*-*GAL4*,*abi*^*5*^/*UAS*-*HA*-*abi*,*Df* (Abi rescue-post). **c**, **d** Loss of Abi causes an increase in NMJ synaptic transmission. **c** Representative two-electrode voltage-clamp (−60 mV) records from muscle 6 with 0.5 Hz nerve stimulation. Sample excitatory junction current (EJC) traces are shown for wild type, *abi*^*5*^/*Df*, *abi*-*GAL4*/+ transgenic control, and *abi*-*GAL4*/+; *UAS*-*HA*-*abi*,*Df*/*abi*^*5*^ (*abi*-*GAL4* rescue). **d** Quantification of EJC amplitude. **e** Schematic view of the domain structure of wild-type Abi and Abi variants used in rescue experiments. WAB, WAVE-binding domain; HHR, homeodomain homologous region; P, proline-rich region; SH3, Src homology 3 domain. Point mutations are indicated by stars. Bar graphs show mean ± s.e.m. Sample number analyzed is indicated inside bars. Statistical analyses were performed by one-way analysis of variance with Tukey–Kramer post hoc test. Comparisons are with wild type (*P < 0.001; **P < 0.01; ***P < 0.05)

Null *abi* mutants display NMJ overgrowth with supernumerary boutons, including excessive formation of immature satellite boutons^14^. This *abi*^*5*^/*Df* phenotype is observed at every NMJ, including NMJ 6/7 and NMJ 4 (Fig. 2a). Compared with genetic controls (*w*^*1118*^), synaptic bouton number at the NMJ 6/7 terminal is increased on average by ~40% in the absence of Abi. With normalization to muscle surface area, bouton number remains ~40% larger in *abi*^*5*^/*Df* compared to matched controls (Fig. 2b). Null *abi* mutants display an even greater elevation in satellite boutons (Fig. 2a, arrowheads), with *abi*^*5*^/*Df* mutants showing a ~90% increase at the NMJ 6/7 terminal (Fig. 2b). Satellite boutons in *abi*^*5*^/*Df* mutants are surrounded by SSR marker Dlg, with presynaptic active zone Bruchpilot (Brp) juxtaposed to postsynaptic glutamate receptor (GluRIIC) domains (Supplementary Fig. 1). Thus satellite boutons in *abi* mutants display the predicted features of a functional bouton. To test whether Abi is required presynaptically or postsynaptically, *UAS*-*HA*-*abi* was expressed in *abi*^*5*^/*Df* mutants using the UAS/GAL4 system. Expression in neurons using the *C155*-*GAL4* driver completely rescues synaptic defects in *abi* null mutants, whereas expression in the muscles using *24B*-*GAL4* fails to provide any rescue (Fig. 2a, b). Thus Abi is required in the neuron to regulate growth and structural architecture at the NMJ synapse.

To assay functional neurotransmission strength in *abi* mutants, evoked excitatory junction current (EJC) recordings were made in two-electrode voltage-clamp (TEVC) configuration. Null *abi* mutants (*abi*^*5*^/*Df*) display clearly increased evoked EJC amplitudes (Fig. 2c). Compared with wild-type controls, the mean EJC amplitude at the NMJ 6/7 is increased by 20% in *abi* null mutants (*w*^*1118*^: 224 ± 11.9 nA; *abi*^*5*^/*Df*: 269 ± 7.4 nA; *P* < 0.05; Fig. 2c, d). Expression of wild-type *UAS*-*HA*-*abi* under the control of endogenous *abi*-*GAL4* is sufficient to decrease the mean EJC amplitude by 25% in the *abi*^*5*^/*Df* mutant background relative to the transgenic control (*abi*-*GAL4*/+: 203 ± 12.2 nA; *abi*-*GAL4* rescue: 153 ± 8.1 nA; *P* < 0.01; Fig. 2c, d). Indeed, the elevated transgenic expression of *abi* suppresses EJC neurotransmission strength significantly below the control level, showing that the *abi* dosage bidirectionally determines synapse function under both loss- and gain-of-expression conditions (Fig. 2c, d). These results indicate that loss of Abi causes an increase in NMJ synaptic transmission and indicate the importance of the precise level of Abi for determining normal neurotransmission strength.

### Abi domain requirements in synaptic development regulation

Abi is a multi-modular protein with N-terminal SCAR/WAVE-binding (WAB) domain, central Kette/NAP1-binding homeodomain homologous region (HHR), and C-terminal Src homology 3 (SH3) domain, which interacts with the Abl tyrosine kinase and WASp (Supplementary Fig. 2a)^25,29,41^. Abl phosphorylates multiple Abi tyrosine residues, including Y148, Y155, Y248, and Y285 (ref. ^28^). To investigate Abi domain requirements, we performed rescue experiments in *abi* null mutants using transgenes for SCAR-binding defective HA-AbiΔ30–65, Kette-binding defective HA-AbiΔ123–175, WASp-binding defective HA-Abi-W452K, phospho-mimic HA-Abi-4YE (Y148E + Y155E + Y248E + Y285E), and phospho-defective HA-Abi-4YF (Y148F + Y155F + Y248F + Y285F) (Fig. 2e; see Supplementary Note 1 and Supplementary Fig. 2b for the characterization of Abi mutants). Neuronal expression of HA-Abi-W452K or HA-Abi-4YE fully rescues the *abi* null NMJ phenotypes (Fig. 2b). Both the number of mature synaptic boutons (top) and immature satellite boutons (bottom) are indistinguishable from control. In contrast, the HA-AbiΔ30–65, HA-AbiΔ123–175, and HA-Abi-4YF lines all fail to exhibit any rescue activity (Fig. 2b). These three conditions show the same increase in synaptic bouton number and supernumerary satellite bouton formation that characterizes the *abi* null mutant. Importantly, expression levels and presynaptic targeting of all transgenes are indistinguishable from those of wild-type HA-Abi transgene (Supplementary Fig. 2c, d). Taken together, these results suggest that Abl-mediated phosphorylation of Abi and its interactions with SCAR and Kette, but not with WASp, are all required for the proper regulation of synaptic development.

### Abi links Abl function and Rac1/SCAR signaling at the NMJ

**Fig. 3:**
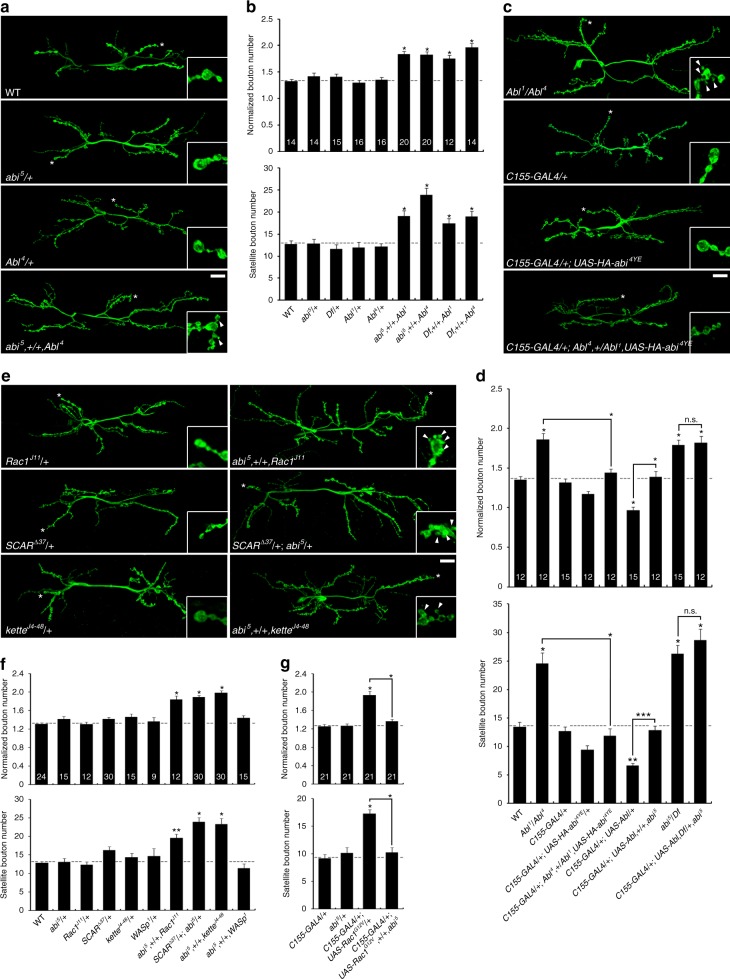
Abi acts downstream of Abl and Rac1 via SCAR complex activation. **a**–**d** Genetic interactions between *abi* and *Abl* regulating synaptic architecture. **a** Sample confocal images of anti-HRP-stained neuromuscular junction (NMJ) 6/7 in wild type, *abi*^*5*^/+, *Abl*^*4*^/+, and *abi*^*5*^,+/+,*Abl*^*4*^. **b** Quantification of total (top) and satellite (bottom) bouton number reveals a transheterozygous interaction between *abi* and *Abl*. **c** Sample confocal images of anti-HRP-stained NMJ 6/7 in *Abl*^*1*^/*Abl*^*4*^, *C155*-*GAL4*/+, *C155*-*GAL4*/+; *UAS*-*HA*-*abi*^*4YE*^, and *C155*-*GAL4*/+; *Abl*^*4*^,+/*Abl*^*1*^,*UAS*-*HA*-*abi*^*4YE*^. **d** Quantification of total (top) and satellite (bottom) bouton number reveals that *Abl* synaptic overgrowth is suppressed by neural overexpression of phospho-mimic Abi-4YE, while Abl-induced synaptic undergrowth is suppressed by decreasing *abi* gene dose. **e**–**g** Genetic interactions between *abi* and *Rac1*, *SCAR* and *kette*. **e** Sample confocal images of anti-HRP-stained NMJ 6/7 in *Rac1*^*J11*^/+, *SCAR*^*Δ37*^/+, *kette*^*J4–48*^/+, *abi*^*5*^,+/+,*Rac1*^*J11*^, *SCAR*^*Δ37*^/+; *abi*^*5*^/+, and *abi*^*5*^,+/+,*kette*^*J4–48*^. **f** Quantification of total (top) and satellite (bottom) bouton number reveals transheterozygous interactions of *abi* with *Rac1*, *SCAR*, and *kette* but not with *WASp*. **g** Quantification of total (top) and satellite (bottom) bouton number reveals that Rac1-G12V-induced overgrowth is suppressed by decreasing *abi* gene dose. Bar graphs indicate mean ± s.e.m. NMJ number analyzed for each genotype is indicated inside bars. Comparisons made against wild type (**b**, **d**, **f**) or *C155*-*GAL4*/+ (**g**) unless indicated (one-way analysis of variance with Tukey–Kramer post hoc test; *P < 0.001; **P < 0.01; ***P < 0.05; n.s. not significant). Insets (**a**, **c**, **e**) show higher magnification views of boutons marked by white asterisks. Satellite boutons are indicated by arrowheads. Scale bars: 20 μm

The Abi structure–function analyses suggest Abl and the Rac1-SCAR pathway function with Abi at the synapse. To further test the Abl–Abi link, we first assayed whether Abl is required in synaptic development. *Abl* nulls (*Abl*^*1*^/*Abl*^*4*^) and targeted expression of dominant-negative, kinase-dead Abl (Abl-K417N) in neurons increase total and satellite bouton numbers (Supplementary Figs. 1 and 3a, b), mimicking *abi* loss of function. In contrast, postsynaptic expression of Abl-K417N does not alter synaptic development, supporting a presynaptic role. Given the similar *abi* and *Abl* phenotypes, we next examined genetic interactions between *abi* and *Abl*. Larvae carrying one null copy of *abi* and one null copy of *Abl* display synaptic overgrowth with excessive satellite bouton formation (Fig. 3a, b), with no defects in either heterozygous mutation alone, suggesting that Abi and Abl act in a common pathway. We next pursued two additional experiments to test whether *abi* is epistatic to *Abl*. Neuronal expression of HA-Abi-4YE, which has no effect on synaptic development by itself, completely suppresses the synaptic defects of *Abl* mutants (Fig. 3c, d). Moreover, neuronal expression of wild-type Abl induces synaptic undergrowth, and this defect is completely suppressed by removing one copy of *abi* (Fig. 3d). However, neuronal expression of Abl fails to suppress synaptic overgrowth in *abi* mutants (Fig. 3d). These results show that Abi functions downstream of Abl during synaptic development.

We next further tested the functional link between Abi and the Rac1-SCAR pathway. Neuronal expression of dominant-negative Rac1-T17N^42^, *SCAR*^*RNAi*^, or *kette*^*RNAi*^ increases total synaptic bouton number as well as satellite bouton number (Supplementary Figs. 1 and 3c, d), recapitulating the phenotypes of *abi* mutants. In addition, we find similar phenotypes in larvae transheterozygous for *abi* and *Rac1*, *SCAR*, or *kette*, but we never observe any synaptic defects in single heterozygotes alone (Fig. 3e, f). However, *abi* null mutants fail to show any heterozygous interaction with *WASp* (Fig. 3f), demonstrating the specificity of the SCAR complex compared to the WASp complex in the Abi-mediated regulation of synaptic development. Neuronal expression of constitutively active Rac1-G12V^42^ also induces synaptic overgrowth with excessive satellite bouton formation as previously reported^10^. These synaptic phenotypes are also fully suppressed by removing a single copy of *abi* (Fig. 3g). Finally, neuronal expression of HA-Abi or HA-Abi-YE fails to suppress the synaptic overgrowth caused by Rac1-T17N, *SCAR*^*RNAi*^, or *kette*^*RNAi*^ expression (Supplementary Fig. 3d). Taken together, these results support the conclusion that Abi acts exclusively through the Rac1-SCAR pathway.

### Abi, Abl, and Rac1 regulate the BMP-dFMRP-Futsch pathway

**Fig. 4:**
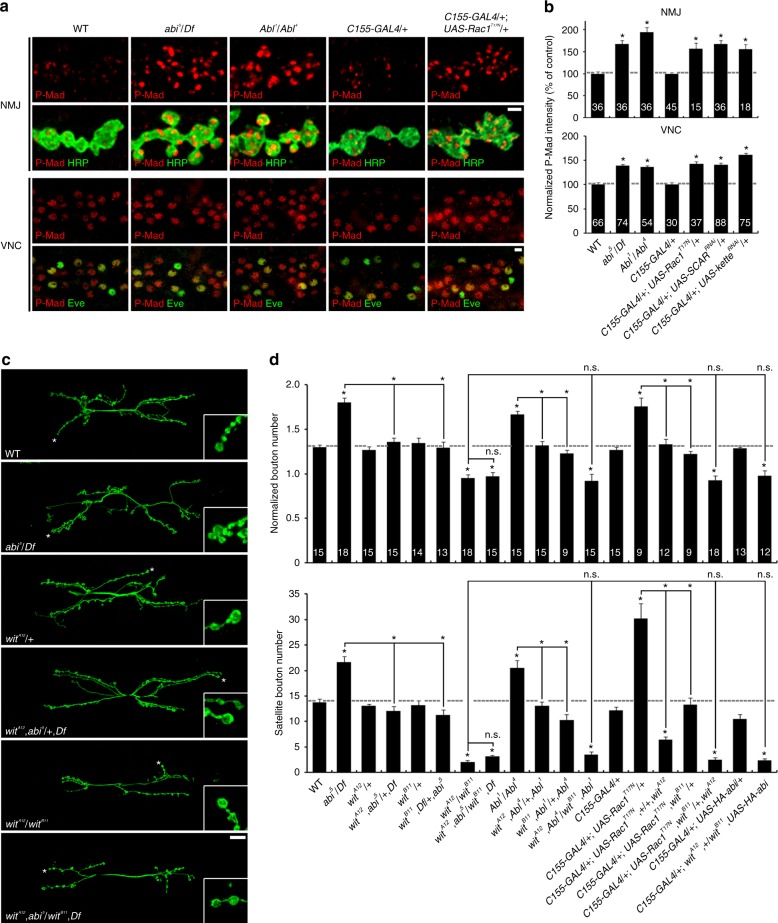
Abi pathway restrains synaptic development by inhibiting bone morphogenetic protein (BMP) signaling. **a**, **b** Loss of Abi, Abl, and Rac1-SCAR components increases P-Mad in motor neurons. **a** Single confocal sections of neuromuscular junction (NMJ) 6/7 (top) and ventral nerve cord (VNC) co-labeled with anti-P-Mad and anti-HRP (NMJ) or anti-Even Skipped (Eve; VNC) shown for wild type, *abi*^*5*^/*Df*, *Abl*^*1*^/*Abl*^*4*^, *C155*-*GAL4*/+, and *C155*-*GAL4*/+; *UAS*-*Rac1*^*T17N*^/+. **b** Quantification of P-Mad intensity normalized to HRP or Eve in the indicated genotypes. **c**, **d** Synaptic overgrowth induced by loss of Abi, Abl, or presynaptic Rac1 depends on BMP signaling. **c** Sample confocal images of anti-HRP-labeled NMJs in wild type, *abi*^*5*^/*Df*, *wit*^*A12*^/+, *wit*^*A12*^,*abi*^*5*^/+,*Df*, *wit*^*A12*^/*wit*^*B11*^, and *wit*^*A12*^,*abi*^*5*^/*wit*^*B11*^,*Df*. **d** Quantification of synaptic structure at NMJ 6/7 in the indicated genotypes. Bar graphs indicate mean ± s.e.m. The number of NMJ branches (**b**, upper panel), Eve-positive motor neurons (**b**, bottom panel), or NMJs (**d**) analyzed for each genotype is indicated inside bars. All comparisons are with wild type (**b**, **d**) unless indicated (one-way analysis of variance with Tukey–Kramer post hoc test; *P < 0.001; n.s. not significant). Scale bars: **a**, 2 μm (NMJ); **a**, 5 μm (VNC); **c**, 20 μm

Elevated *trans*-synaptic BMP signaling is a well-established mechanism underlying synaptic expansion and bouton formation^11,13,43^. We therefore hypothesized that the synaptic overgrowth caused by loss of Abi, Abl, Rac1, or components of SCAR complex might be due to increased retrograde BMP signaling. To test this hypothesis, we first examined the BMP-dependent production of phosphorylated Mad (P-Mad) in the presynaptic terminal and accumulation in neuronal nuclei, as established molecular readouts of BMP *trans*-synaptic signaling activity^5,7^. P-Mad levels are significantly higher in both *abi* and *Abl* mutants compared to matched controls, both within NMJ synaptic boutons and in the downstream ventral nerve cord (VNC) motor neuron nuclei (Fig. 4a, b). Neuronal expression of Rac1-T17N, *SCAR*^*RNAi*^, or *kette*^*RNAi*^ similarly produces the same elevated P-Mad expression both in the NMJ presynaptic terminal and within the VNC motor neuron nuclei (Fig. 4a, b). These results suggest that Abi, Abl, Rac1, and the SCAR complex all negatively regulate BMP signaling during synaptic development.

We next examined genetic interactions of *abi*, *Abl*, and *Rac1* with the BMP type II receptor *wishful thinking* (*wit*)^6,7^. Removal of one copy of *wit* alone has no detectable effect on synaptic development but significantly suppresses the synaptic overgrowth of *abi*^*5*^/*Df* null mutants (Fig. 4c, d). With reduced BMP signaling, both the elevated number of mature synaptic boutons (top) and excessive immature satellite boutons (bottom) decrease toward control levels. Moreover, removing both copies of *wit* further suppresses the synaptic defects in *abi* mutants; *wit*, *abi* double mutants are not detectably different from *wit* single mutants (Fig. 4c, d), which show significant synaptic undergrowth^6,7^. Once again, the dependence on BMP signaling is observed for both total synaptic bouton number and satellite bouton formation. Similar dosage-sensitive genetic interactions occur between *wit* and either *Abl* or *Rac1* (Fig. 4d), showing that these phenotypes also depend on BMP signaling. Finally, neuronal overexpression of *UAS*-*HA*-*abi* fails to suppress the synaptic undergrowth phenotype of *wit*-null mutants (Fig. 4d). These findings suggest that Abi, Abl, and Rac1-SCAR signaling restrains synaptic development via inhibition of BMP *trans*-synaptic signaling.

We have previously shown that BMP signaling promotes NMJ synaptic growth by repressing the expression of *Drosophila* Fragile X Mental Retardation Protein (dFMRP)^11^, which in turn inhibits Futsch/MAP1B translation^12^. BMP-mediated maintenance of microtubule (MT) stability via the dFMRP-Futsch pathway is critical for synapse regulation. As detailed in Supplementary Note 2 and Supplementary Fig. 4, we find that the Abi-SCAR complex acts downstream of Abl and Rac1 to regulate synaptic development via the BMP-dFMRP-Futsch-MT stability pathway in the presynaptic terminal.

### Abi acts downstream of Abl in Rac-mediated macropinocytosis

We next tested how Abi regulates BMP *trans*-synaptic signaling. Previous studies show that BMPR endocytosis is a key regulatory mechanism^44^. Moreover, mammalian Abi, Rac1, and SCAR are all essential for signaling-induced macropinocytosis, at least in non-neuronal cells^18,45^. We therefore hypothesized Abi might function downstream of Abl in a Rac1-SCAR pathway controlling macropinocytosis to inhibit *trans*-synaptic BMP signaling. To test this idea, we first characterized macropinocytosis in *Drosophila* BG2-c2 neuronal cells using a classic macropinocytic tracer; dextran (70 kDa) conjugated to tetramethylrhodamine (TMR-Dex). As detailed in Supplementary Note 3 and Supplementary Fig. 5, uptake of TMR-Dex by BG2-c2 cells occurs at background levels under basal conditions but is potently induced by Gbb stimulation in a manner dependent on Abi, Abl, Rac1, SCAR, and Kette. Gbb stimulation also induces Abi-dependent formation of F-actin-rich membrane ruffles (Supplementary Note 3 and Supplementary Fig. 5h), which are functionally coupled to macropinocytosis^16^. 

**Fig. 5:**
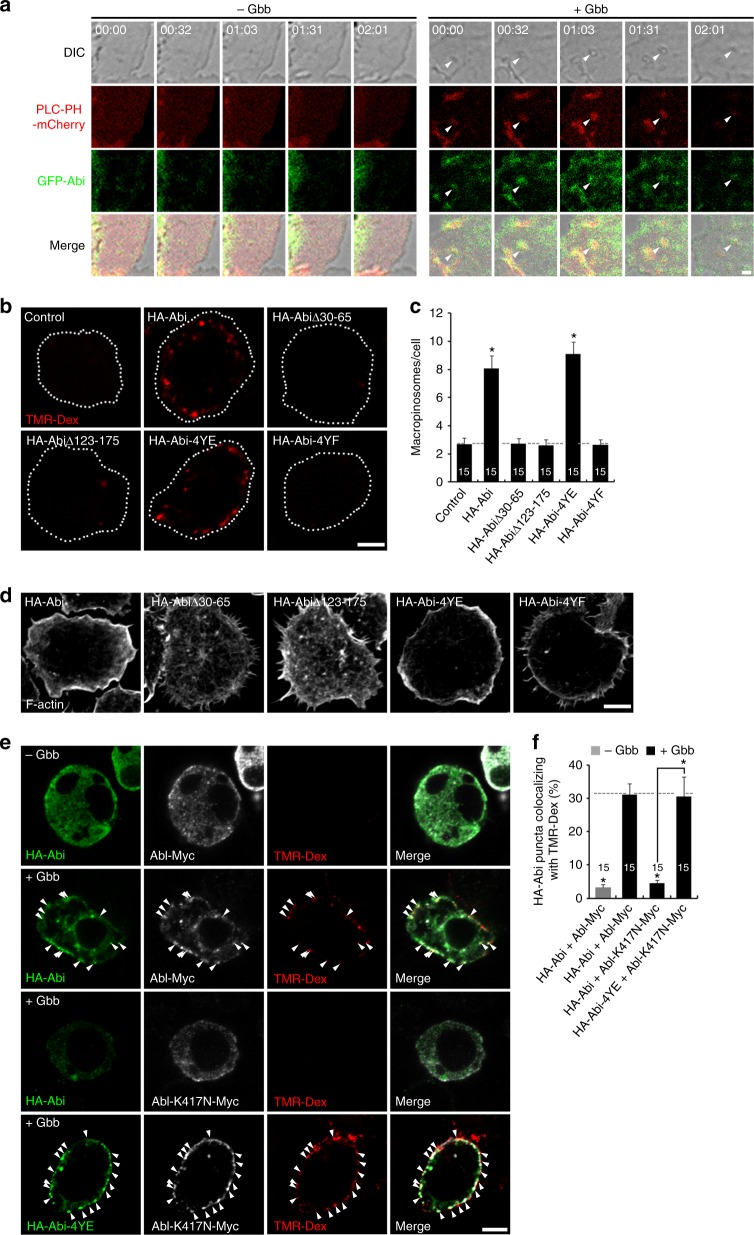
Abi domain requirements in Gbb-induced early macropinocytosis. **a** Live, time-lapse imaging of control or Gbb (50 ng/ml)-stimulated BG2-c2 cells expressing PI(4,5)P_2_ probe PLC-PH-mCherry and GFP-Abi. Differential interference contrast images show elapsed time (min:s) in upper panels. Arrowheads mark a forming macropinosome. **b** Confocal images of BG2-c2 cells transfected with dsRNA for 3′ untranslated region of *abi* alone (control) or in combination with HA-Abi, HA-AbiΔ30–65, HA-AbiΔ123–175, HA-Abi-4YE, or HA-Abi-4YF, serum starved for 6 h, and pulsed with 2 mg/ml TMR-Dex in the presence of 50 ng/ml Gbb for 5 min. **c** Quantification of TMR-Dex-filled macropinosomes (puncta >0.2 μm in diameter). **d** Single confocal sections of cells treated as in **b** and labeled for F-actin with rhodamine-phalloidin. **e** Single confocal sections of cells expressing HA-Abi or HA-Abi-4YE together with Abl-Myc or Abl-K417N-Myc, incubated for 2 min with 2 mg/ml TMR-Dex and Gbb (50 ng/ml), and then labeled with anti-HA and anti-Myc. Arrowheads show HA-Abi-positive and Abl-Myc-positive puncta containing TMR-Dex. **f** Quantification of HA-Abi-TMR-Dex colocalization. Bar graphs indicate mean ± s.e.m. The number of cells analyzed is indicated inside bars. Comparisons are made against control cells transfected with *abi* dsRNA (**c**) or Gbb-stimulated cells expressing HA-Abi and Abl-Myc unless indicated (one-way analysis of variance with Tukey–Kramer post hoc test; *P < 0.001). Scale bars: **a**, 1 μm; **b**, **d**, **e**, 5 μm

We next examined the association of Abi with macropinocytic structures using the phospholipase Cδ1-pleckstrin homology domain-mCherry (PLC-PH-mCherry) reporter of PI(4,5)P_2_ (ref. ^46^), which labels membrane ruffles and macropinocytic cups, the precursors of macropinosomes^47^. Live, time-lapse imaging shows green fluorescent protein (GFP)-Abi associates with Gbb-induced, PLC-PH-mCherry-positive membrane ruffles and macropinocytic cups (Fig. 5a). As detailed in Supplementary Note 3, immunostaining of cells uptaken TMR-Dex in the presence of Gbb and TMR-Dex pulse-chase experiments reveal that Abi is also transiently localized on newly formed macropinosomes (Supplementary Figs 5i and 6). These results support a critical role of Abi in macropinosome formation.

We next tested relationships between Abi, Abl, and Rac1-SCAR during Gbb-induced macropinocytosis. Expression of wild-type HA-Abi or phospho-mimic HA-Abi-4YE in Abi-knockdown cells fully restores Gbb-induced membrane ruffling and TMR-Dex uptake to control levels (Fig. 5b–d). In contrast, SCAR-binding mutant HA-AbiΔ30–65, Kette-binding mutant HA-AbiΔ123–175, and phospho-defective mutant HA-Abi-4YF all fail to function (Fig. 5b–d). These results show that phosphorylation of Abi and its interactions with both SCAR and Kette are required for Gbb-induced macropinocytosis. Previous work has shown that Abl-mediated phosphorylation of Abi regulates subcellular localization^28^. To test whether Abl is needed for Abi targeting to macropinocytic structures, BG2-c2 cells coexpressing HA-Abi and Abl-Myc were incubated with TMR-Dex in the absence or presence of Gbb (Fig. 5e, f). Unstimulated controls show low levels of uptake and diffuse Abi cytoplasmic distribution. In contrast, Gbb strongly induces uptake and recruitment of HA-Abi and Abl-Myc to peripheral punctate structures containing internalized TMR-Dex (Fig. 5e, f). These changes are not observed in cells coexpressing kinase-dead Abl-K417N-Myc. Importantly, Gbb-induced TMR-Dex uptake and Abl/Abi recruitment are observed with HA-Abi-4YE and Abl-K417N-Myc coexpression (Fig. 5e, f). These results show that Abl-mediated phosphorylation of Abi is critical for its recruitment to forming macropinosomes.

### Gbb-induced macropinocytosis required for BMPR degradation

**Fig. 6:**
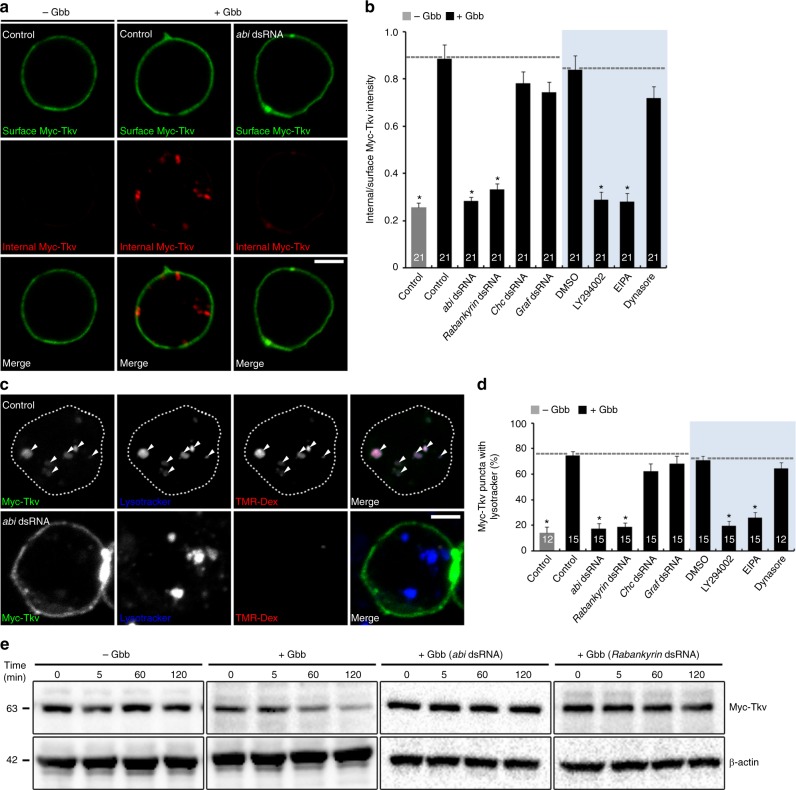
Abi-dependent macropinocytosis in Gbb-induced Tkv degradation. **a** Gbb induces macropinocytosis-mediated internalization of Tkv receptor. Single confocal slices of BG2-c2 cells transfected with Myc-Tkv alone (control) or together with *abi* dsRNA. Cells were serum-starved for 6 h, prelabeled with anti-Myc antibody at 4 °C for 30 min, and then incubated in the absence (− Gbb) or presence (+ Gbb) of Gbb at 25 °C (50 ng/ml, 5 min). After fixing, surface and internalized receptors were sequentially fluorescent-labeled with secondary antibodies under nonpermeant (green) and permeant (red) conditions, respectively. **b** Quantification of internal-to-surface Myc-Tkv ratio in controls versus cells transfected with the indicated dsRNA. Shading: BG2-c2 cells were pretreated with dimethyl sulfoxide (DMSO; 0.1%), LY294002 (25 μM), 5-*N*-ethyl-*N*-isopropyl-amiloride (100 μM), or dynasore (20 μM) for 30 min. **c** Macropinocytosis of internalized receptors in BG2-c2 cells transfected with Myc-Tkv alone (control) or together with *abi* dsRNA. Serum-starved cells prelabeled with anti-Myc antibody (4 °C, 30 min) were pulsed with TMR-Dex (70 kDa, 2 mg/ml) in the absence (− Gbb) or presence (+ Gbb) of Gbb at 25 °C (50 ng/ml, 2 min) and then chased for 18 min. Cells were labeled with Lysotracker (1 μM, 5 min) prior to anti-Myc staining. Single confocal sections through the middle of the cells are shown. **d** Quantification of Myc-Tkv-Lysotracker colocalization in cells of the indicated genotypes. **e** Macropinocytosis mediates ligand-induced Tkv degradation. Western blot analysis of total BG2-c2 cell extracts transfected with Myc-Tkv alone or together with the indicated dsRNA. Serum-starved cells were pretreated with cycloheximide (50 μg/ml, 3 h) to inhibit new protein synthesis and then stimulated with Gbb (50 ng/ml) at 25 °C for the indicated times. Bar graphs indicate mean ± s.e.m. The number of cells analyzed for each genotype is indicated inside bars (**b**, **d**). All comparisons are made against Gbb-treated (unshaded) or Gbb- and DMSO-treated (shaded) control (one-way analysis of variance with Tukey–Kramer post hoc test; *P < 0.001). Scale bars: 5 μm

To test whether Gbb-induced macropinocytosis drives BMPR degradation, we used an antibody-feeding assay in BG2-c2 cells expressing Myc-Tkv or Myc-Wit. This experiment prelabels surface receptors with anti-Myc antibody (4 °C) to quantify both surface and internalized pools after 5 min in the absence and presence of Gbb (25 °C). In unstimulated cells, receptors internalize at low levels (Fig. 6a and Supplementary Fig. 7a). In contrast, Gbb stimulation causes both Myc-Tkv and Myc-Wit to rapidly incorporate into intracellular punctate structures (Fig. 6a and Supplementary Fig. 7a). The intracellular to surface receptor ratio is potently increased in Gbb-stimulated cells versus unstimulated controls (Fig. 6b and Supplementary Fig. 7b). Gbb-induced uptake is completely abrogated by knockdown of  Abi or the macropinocytosis regulator  Rabankyrin and by pretreatment with the macropinocytosis inhibitor LY294002 or 5-*N*-ethyl-*N*-isopropyl-amiloride (EIPA) (Fig. 6b). In contrast, receptor internalization is not impaired by knockdown of clathrin heavy chain (Chc) or GTPase regulator associated with focal adhesion kinase (Graf)^48^ driving endocytosis, demonstrating a selective role for macropinocytosis in BMPR internalization. Consistent with macropinocytosis being dynamin independent^49^, the dynamin inhibitor dynasore also does not affect the Gbb-induced uptake of receptors (Fig. 6b). These results suggest that Gbb stimulation drives BMPR internalization via macropinocytosis.

We next combined antibody feeding with a TMR-Dex pulse-chase assay. BG2-c2 cells prelabeled for Myc-Tkv or Myc-Wit surface receptors were TMR-Dex pulsed with Gbb stimulation (2 min) and then chased (3 min). Internalized receptors colocalize (69–76%) with TMR-Dex-positive macropinosomes (Supplementary Fig. 7c–e). We tested sorting of internalized Myc-Tkv receptors to lysosomes at 23 min and found a complete colocalization between internalized Myc-Tkv and TMR-Dex in punctate structures (Fig. 6c). Notably, most (~75%) of the Myc-Tkv colocalizes with the lysosome marker Lysotracker (Fig. 6d). Internalized Myc-Tkv and TMR-Dex are significantly diminished by depleting Abi or Rabankyrin, as well as by pretreating cells with LY294002 or EIPA. Importantly, interfering with macropinocytosis also greatly reduces the extent of Myc-Tkv/Lysotracker colocalization (Fig. 6d). In contrast, colocalization is not affected by either depleting Chc or Graf or pretreating dynasore (Fig. 6d). We also directly tested the impacts of Gbb stimulation and Abi knockdown on Myc-Tkv receptor degradation by western blot analysis. Constitutive receptor degradation is minimal in the absence of Gbb stimulation (Fig. 6e). In contrast, Myc-Tkv is robustly degraded in Gbb-stimulated cells over a 2-h period in a mechanism blocked by both Abi and Rabankyrin knockdown (Fig. 6e). These results suggest that Gbb-induced macropinocytosis efficiently commits BMPRs to degradation.

### Abi-dependent macropinocytosis downregulates synaptic BMPRs

**Fig. 7:**
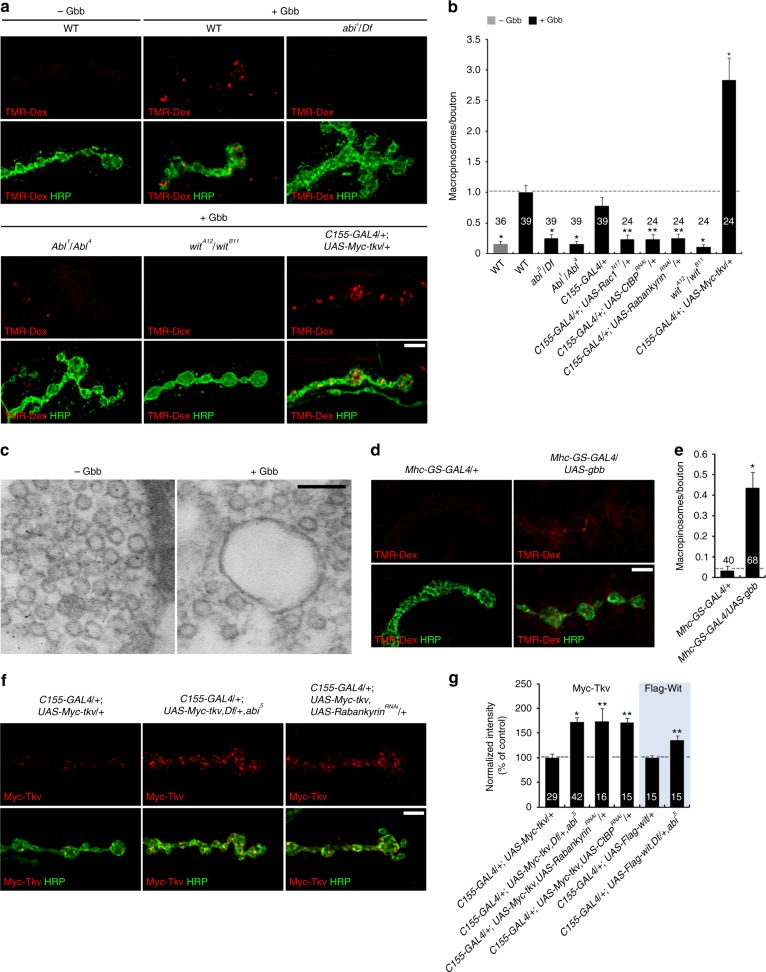
Synaptic macropinocytosis-mediated downregulation of Tkv receptors. **a** Stimulation of neuromuscular junction (NMJ) macropinocytosis by recombinant Gbb. Sample confocal images of NMJ 6/7 boutons in the indicated genotypes labeled with anti-HRP (green) following pulse of TMR-Dex (70 kDa, 2 mg/ml, red) in both absence (− Gbb) and presence (+ Gbb) of Gbb (50 ng/ml, 5 min). **b** Quantification of TMR-Dex-positive macropinosomes (>0.2 μm diameter) per NMJ bouton. **c** Representative transmission electron microscopic images of control or Gbb-stimulated NMJ 6/7 boutons overexpressing bone morphogenetic protein receptor (BMPR) Tkv (*C155*-*GAL4*/+; *UAS*-*Myc*-*tkv*/+). **d** Macropinocytosis stimulation from postsynaptic Gbb driven by *Mhc*-*GS*-*GAL4*/*UAS*-*gbb* in early third instar (RU486 [3 mg/ml], 2 min), aged for 8 h to the late third instar. Sample images of NMJ 6/7 boutons from *Mhc*-*GS*-*GAL4*/+ (control) and *Mhc*-*GS*-*GAL4*/*UAS*-*gbb* labeled with anti-HRP (green) following pulse of TMR-Dex (2 mg/ml, 5 min, red). **e** Quantification of TMR-Dex-positive macropinosomes/NMJ bouton. **f** Single confocal sections of NMJ 6/7 boutons of the indicated genotypes stained with anti-HRP (green) and anti-Myc (red) under permeant conditions. **g** Quantification of BMPR intensities. Bar graphs indicate mean ± s.e.m. The number of NMJ branches analyzed for each genotype is indicated inside bars. Statistical analyses were performed by one-way analysis of variance with Tukey–Kramer post hoc test for **b** and **g** (Myc-Tkv) or by Student’s *t* test for **e** and **g** (Flag-Wit). Comparisons are made against Gbb-stimulated wild type (**b**), *Mhc*-*GS*-*GAL4*/+ (**e**), *C155*-*GAL4*/+; *UAS*-*Myc*-*tkv*/+ (**g**), or *C155*-*GAL4*/+; *UAS*-*Flag*-*wit*/+ (**g**, shaded) NMJs (*P < 0.001; **P < 0.01). Scale bars: **a**, **d**, **f**, 5 μm; **c**, 100 nm

We next tested Gbb-induced, Abi-dependent macropinocytosis in BMP regulation at the NMJ. Since macropinocytosis has not been reported at any synapse, we first tested whether macropinocytosis is active. Acutely dissected third instar larvae were pulse-labeled with TMR-Dex for 5 min to assay for dye uptake. Wild-type NMJs show little TMR-Dex incorporation (Fig. 7a, b). In sharp contrast, however, application of Gbb (50 ng/ml) strongly induces TMR-Dex uptake in large, internalized vesicles (Fig. 7a, b). This Gbb-induced uptake is completely abrogated by loss of Abi, Abl, Rac1, and two macropinocytosis regulators, Rabankyrin and C-terminal-binding protein (CtBP^49^; Fig. 7b). These results demonstrate Gbb-induced and Abi/Abl/Rac1-mediated macropinocytosis at the NMJ. Importantly, this endocytosis mechanism is greatly inhibited by loss of BMPR and strongly enhanced by presynaptic overexpression of BMPR (Fig. 7a, b). To verify macropinosomes after Gbb induction, we assayed the synaptic ultrastructure of Gbb-stimulated and unstimulated boutons overexpressing Tkv. Transmission electron microscopic measurements were done in NMJ 6/7 synaptic boutons, employing a macropinosome definition of 200 nm in diameter^50^. Unstimulated boutons display uniform synaptic vesicles with 30–40 nm diameters (Fig. 7c). Gbb-stimulated boutons additionally show large, clear macropinosomes with diameters exceeding 200 nm (Fig. 7c). These results indicate that Gbb induces synaptic macropinocytosis through BMPR activation.

We next tested whether NMJ macropinocytosis is induced by postsynaptic Gbb release by employing the muscle-specific *Mhc*-*GeneSwicth*-*GAL4* (*Mhc*-*GS*-*GAL4*) driver, which induces conditional transgene expression in an RU486-dependent fashion^51^. Third instar *Mhc*-*GS*-*GAL4*/*UAS*-*gbb* fed RU486 and aged for 8 h show elevated Gbb release and consequent NMJ overgrowth (Supplementary Fig. 8a–d). Importantly, this elevated postsynaptic Gbb expression causes strong stimulation of macropinocytosis in presynaptic boutons (Fig. 7d, e). These results suggest that presynaptic macropinocytosis can be driven under physiological conditions by an increase of postsynaptic Gbb release. In parallel, we also examined localization of presynaptic Myc-Tkv in NMJ boutons to test for roles of macropinocytosis in internalizing BMPRs. We find partial colocalization of the Myc-Tkv puncta with HA-Abi, GFP-Rabankyrin, and CtBP-GFP (HA-Abi: 46.1%, *n* = 24; GFP-Rabankyrin: 48.6%, *n* *=* 22; CtBP-GFP: 55.1%, *n* = 29; Supplementary Fig. 8e), suggesting that presynaptic BMPRs are internalized via Abi-dependent macropinocytosis. We also assessed steady-state levels of neuronally expressed Myc-Tkv and Flag-Wit at the NMJ to show that both total and surface levels of both synaptic receptors are significantly increased by Abi, Rabankyrin, or CtBP loss (Fig. 7f, g and Supplementary Fig. 8f, g). These results strongly support a role for Abi-dependent macropinocytosis in the downregulation of presynaptic BMPRs. However, Abi-dependent macropinocytosis is not required for efficient synaptic vesicle recycling during depolarizing stimulation as detailed in Supplementary Note 4.

### Macropinocytosis regulation of NMJ synaptic development

**Fig. 8:**
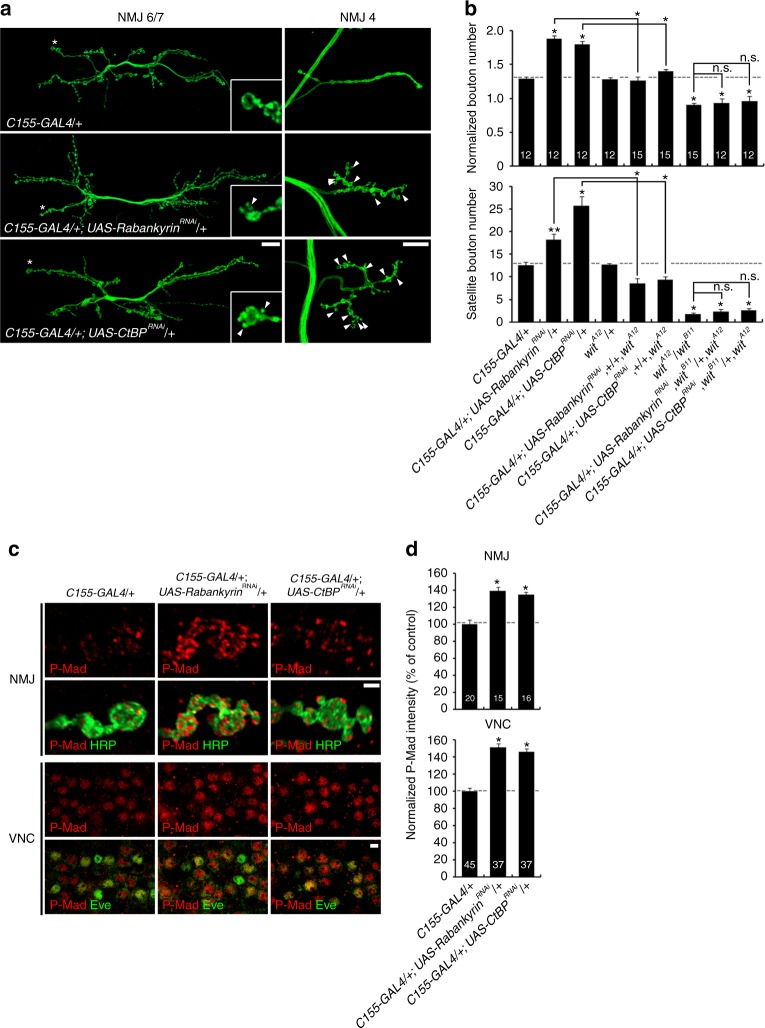
Rabankyrin and C-terminal-binding protein (CtBP) inhibit bone morphogenetic protein (BMP)-dependent synaptic development. **a** Presynaptic Rabankyrin or CtBP RNA interference (RNAi) induces BMP-dependent synaptic overgrowth. Sample images of anti-HRP-labeled neuromuscular junctions (NMJs) 6/7 and 4 in the indicated genotypes. Insets show high-magnification boutons marked by asterisks. Satellite boutons are indicated by arrowheads. **b** Quantification of total (top) and satellite (bottom) bouton number at NMJ 6/7. Note that synaptic overgrowth with Rabankyrin or CtBP knockdown is sensitive to *wit* level. **c** Presynaptic Rabankyrin or CtBP RNAi increases P-Mad in motor neurons. Single confocal sections of NMJ 6/7 (top) and ventral nerve cord (VNC; bottom) co-labeled for anti-P-Mad and anti-HRP (NMJ) or anti-Eve (VNC). **d** Quantification of P-Mad intensity normalized to HRP or Eve. Bar graphs indicate mean ± s.e.m. The number of NMJs (**b**), NMJ branches (**d**, upper panel), or Eve-positive motor neurons (**d**, lower panel) analyzed for each genotype is indicated inside bars. All comparisons against *C155*-*GAL4*/+ unless indicated (one-way analysis of variance with Tukey–Kramer post hoc test; *P < 0.001; **P < 0.01; n.s. not significant). Scale bars: **a**, 20 μm; **c**, 2 μm (NMJ); **c**, 5 μm (VNC)

The above results all support the model of Abi regulating synaptic development by downregulating surface BMPRs via macropinocytosis. We therefore hypothesized that interfering with synaptic macropinocytosis should be sufficient to phenocopy NMJ defects characterizing *abi* mutants. Consistently, mature bouton number, satellite bouton number, and synaptic P-Mad levels are all increased by presynaptic knockdown of Rabankyrin or CtBP (Fig. 8a–d). Moreover, synaptic Futsch/MAP1B expression increases, and the synaptic overgrowth phenotype caused by Rabankyrin or CtBP knockdown is fully rescued by feeding vinblastine (Supplementary Fig. 9a–c). These results suggest that Rabankyrin/CtBP-mediated macropinocytosis limits synaptic growth by modulating MT stability. Moreover, the synaptic overgrowth caused by Rabankyrin or CtBP knockdown is completely suppressed by removing one copy of *wit*, which does not alter NMJ morphology in the wild-type background (Fig. 8b). Furthermore, removal of both copies of *wit*, which causes severe synaptic undergrowth, further suppresses the synaptic overgrowth caused by Rabankyrin or CtBP knockdown. Overall mature bouton number and satellite bouton number in *wit* mutants with Rabankyrin or CtBP knockdown are not significantly different from those in *wit* single mutants alone (Fig. 8b). These findings support the model that macropinocytosis inhibits synaptic development via inhibition of BMP *trans*-synaptic signaling.

## Discussion

Retrograde BMP signaling is a key instructive regulator of synaptic development in animals ranging from *Drosophila* to mammals^3,4^. At the *Drosophila* NMJ, endocytosis and subsequent lysosomal degradation of presynaptic BMPRs is a critical mechanism attenuating this developmental signaling^11,13–15,44^. However, the molecular mechanism involved in this endocytosis-mediated regulation of BMP signaling has been poorly defined. Here we present extensive evidence indicating that the postsynaptically secreted BMP ligand Gbb induces Abi- and SCAR/WAVE-dependent macropinocytosis at the *Drosophila* NMJ. We establish that this mode of endocytosis trafficking is indispensable for signaling-dependent downregulation of BMPRs at the developing NMJ synapse. Furthermore, we show that Abl and Rac1 act downstream of Gbb activation of BMPRs to regulate synaptic macropinocytosis and synaptic growth via the Abi-SCAR complex. These findings indicate that Gbb-induced and Abi/SCAR-dependent macropinocytosis generates a key negative-feedback mechanism that likely safeguards motor neurons against excessive BMP signaling, thus limiting the extent of ligand-induced synaptic growth. This study is the first demonstration of macropinocytosis at any synapse and the first example of macropinosome-dependent limiting control of cell surface receptors in developmental signaling.

Synaptic morphogenesis is tightly dependent on dynamic turnover and reorganization of the actin cytoskeleton^52,53^. At the *Drosophila* NMJ, the Rac1-SCAR and Cdc42-WASp signaling pathways with major roles in activating the Arp2/3 complex nucleating F-actin polymerization^19^ have been implicated in the proper regulation of synaptic development. Mutant disruption of either pathway causes NMJ overgrowth with excessive satellite bouton formation^32–37,54,55^. Importantly, this synaptic architectural over-elaboration phenotype is recapitulated by elevated BMP signaling from postsynaptic muscle to presynaptic nerve terminal^11,13,56^, suggesting a potential link between presynaptic actin cytoskeleton regulation and the mechanism of BMP-dependent synaptic development. However, it has been unclear whether SCAR/WASp-mediated actin polymerization affects NMJ synaptic morphogenesis directly and/or exerts effects through modulation of the BMP signaling pathway. In this study, we have addressed this question by analyzing the synaptic role of Abi, which regulates the activity of SCAR and WASp signaling by acting as a scaffolding protein^18,19,22,25^. Intriguingly, Abi binds and activates the Abl tyrosine kinase, and *Abl* mutants also display striking NMJ synaptic overgrowth^33^. Thus an important question is whether synaptic development requires crosstalk between Abl kinase and SCAR/WASp signaling pathways mediated through Abi.

We provide multiple lines of evidence indicating Abi acts downstream of Abl to regulate BMP-dependent synaptic growth through SCAR but not WASp. We find that loss of presynaptic Abi causes NMJ overgrowth characterized by hyperbudding and excessive satellite bouton formation. Structure–function analyses of Abi combined with genetic rescue studies indicate that normal synaptic development requires the interactions of Abi with Kette and SCAR but not with WASp. In addition, Abi must be phosphorylated by Abl to activate this signaling cascade. Furthermore, *abi*, *Abl*, *Rac1*, *kette*, and *SCAR* display transheterozygous interactions producing NMJ overgrowth, indicating they function together in NMJ synaptic development. Importantly, defects caused by either *Abl* mutation or presynaptic overexpression of constitutively active Rac1 are suppressed by reducing the *abi* genetic dose, demonstrating the epistatic relationship between *Abl*, *Rac1*, and *abi*. We show that Abl-Abi and Rac1-SCAR pathways negatively regulate Gbb-dependent P-Mad production in motor neurons. We also show that both pathways genetically interact with the dFMRP-Futsch/MAP1B pathway, a core signaling module regulating synaptic development through regulation of MT stability^12^. Consistently, synaptic overgrowth caused by loss of Abi, Abl, Rac1, Kette, and SCAR are all completely suppressed by the MT-severing drug vinblastine. Taken together, this work suggests that Abl and Rac1 signaling converge on the Abi-SCAR complex to control BMP-dependent synaptic development.

In mammalian cells, Rac1 and Abi1-SCAR complex regulate macropinocytosis^17,18^, a mode of clathrin- and dynamin-independent endocytosis. This pathway occurs constitutively in antigen-presenting cells (such as macrophages and dendritic cells) but can be induced by growth factors in other cell types, including fibroblasts and epithelial cells^16^. While very little is known about the physiological relevance of macropinocytosis in neurons, previous work has demonstrated that macropinocytosis operates constitutively in axon growth cones of primary hippocampal neurons, supporting membrane retrieval during axonal outgrowth, but apparently ceasing at the onset of synaptogenesis^57^. In *Drosophila* BG2-c2 neuronal cells and NMJ synapses, we find that bona fide macropinocytosis can be induced by Gbb stimulation and that this induction is abrogated by loss of Abi, Rac1, Kette, and SCAR. Furthermore, we find that synaptic macropinocytosis at the *Drosophila* NMJ is also induced by targeted postsynaptic Gbb expression, establishing the in vivo physiological relevance of Gbb-induced macropinocytosis. Finally, both our Abi structure–function analyses and subcellular Abi localization studies show that the Abi-SCAR complex is essential for actin ruffle formation, as a key event driving macropinosome formation^16^. Taken together, these findings establish an essential role for the Abi-SCAR complex in mediating actin polymerization underlying Gbb-induced synaptic macropinocytosis.

Both loss and gain of Rac1 function induces NMJ overgrowth with excess satellite boutons, with acute Rac1 activation driving membrane ruffle and macropinocytic cup formation and deactivation required for efficient macropinosome closure^17^. Thus macropinocytosis critically depends on dynamic changes in Rac1 activity, and our loss and gain-of-function studies of Rac1 support the notion that Rac1 acts through macropinocytosis to restrain BMP-dependent synaptic growth. Given that Abl acts upstream of Abi, a key question is the role of Abl in macropinocytosis. In mammalian cells, Abl phosphorylation of Sos-1, a Rac1 GEF, drives growth factor-induced and Rac1-dependent membrane ruffling^58^. However, evidence supporting a direct Abl role in macropinocytosis has been lacking. We report here that Abl-mediated phosphorylation of Abi is essential for its recruitment to plasma membrane sites of macropinosome formation. At synapses, endocytic downregulation of BMPRs is a core signal attenuation mechanism^11,13–15^. We find that Gbb drives BMPR internalization and degradation via Abi-mediated macropinocytosis. Moreover, loss of Abi or Rabankyrin impairs Gbb-induced degradation of BMPRs. Consistent with *abi* mutant defects, loss of Rabankyrin or CtBP elevates presynaptic BMP signaling and growth, causing supernumerary satellite bouton formation. These findings establish that Gbb-induced macropinocytosis downregulates BMPRs, with Abi restraining synaptic development via Gbb-induced macropinocytosis.

This developmental signaling mechanism may be endemic to the *Drosophila* NMJ, but precedents occur in several other cellular contexts. For example, both platelet-derived growth factor β receptor and vascular endothelial growth factor receptor 2 utilize macropinocytosis during ligand-induced signaling^59,60^. In future studies, it will be interesting to define the mechanisms by which distinct receptor classes employ macropinocytosis to determine differential signaling outcomes. At the *Drosophila* NMJ, synaptic inputs dramatically expand during development to maintain transmission efficacy with rapidly growing muscles^61^. This homeostatic synaptic regulation critically depends on retrograde BMP signaling from the muscle-derived Gbb^3,5–7^. Intriguingly, this same signaling pathway enhances NMJ synaptic growth in response to increased levels of neuronal activity^62^, providing a challenge for motor neurons to prevent the overstimulation of BMPRs. In this regard, one of our key findings is the requirement for BMPR activation in macropinocytosis-driven receptor internalization and degradation. Given this newly discovered cycling mechanism, synaptic macropinocytosis mediated by the Abi/SCAR signaling network likely underlies the homeostatic regulation maintaining the balanced level of NMJ synaptic bouton formation, both during normal synaptogenesis and with the onset of activity-dependent synaptic plasticity.

## Methods

### Drosophila stocks

*w*^*1118*^ was used as genetic control unless otherwise stated. Two P-element alleles of *abi*, G6718 and G4355, were obtained from GenExel (Republic of Korea). The *abi* null allele *abi*^*5*^ was generated via imprecise excision of G6718. Transgenic flies carrying the following constructs were generated in the *w*^*1118*^ background using standard procedures: *UAS*-*HA*-*abi*, *UAS*-*HA*-*abi*^*Δ30–65*^, *UAS*-*HA*-*abi*^*Δ123–175*^, *UAS*-*HA*-*abi*^*W452K*^, *UAS*-*HA*-*abi*^*4YE*^, *UAS*-*HA*-*abi*^*4YF*^, *abi*-*GAL4*, *UAS*-*Myc*-*tkv*, *UAS*-*Flag*-*wit*, *UAS*-*GFP*-*Rabankyrin*, and *UAS*-*CtBP*-*GFP*. *Df(3R)su(Hw)7* (a deficiency uncovering the *abi* locus), *Abl*^*1*^, *Abl*^*4*^, *Rac1*^*J11*^, *WASp*^*1*^, *wit*^*A12*^, *wit*^*B11*^, *dFmr1*^*Δ50M*^, *kette*^*J4–48*^, *SCAR*^*Δ37*^, *shi*^*ts1*^, *UAS*-*Abl*, *UAS*-*Abl*^*K417N*^, *UAS*-*dFmr1*, *UAS*-*Rac1*^*T17N*^, *UAS*-*Rac1*^*G12V*^, *UAS*-*SCAR*^*RNAi*^, *UAS*-*kette*^*RNAi*^, *UAS*-*Rabankryin*^*RNAi*^, and *UAS*-*CtBP*^*RNAi*^ flies were obtained from the Bloomington Stock Center (Supplementary Table 1), and *UAS*-*gbb* from Kristi Wharton (Brown University, USA). The following GAL4 strains were used to drive UAS transgenes: *abi*-*GAL4*, *24B*-*GAL4* (ref. ^63^), *C155*-*GAL4* (Lin and Goodman, 1994), *da*-*GAL4* (ref. ^64^), and *Mhc*-*GeneSwitch*-*GAL4* (*Mhc*-*GS*-*GAL4*)^51^. Animals were cultured on standard medium at 25 °C. For manipulation of MT stability, animals were grown on standard medium containing 1 μM vinblastine sulfate (Sigma-Aldrich). For Gene-Switch experiments, third instar larvae were fed with 3 mg/ml RU486 (Sigma-Aldrich) for 2 min.

### Cloning and molecular analysis

A full-length *abi* cDNA clone was obtained from the *Drosophila* Genomics Resource Center (DGRC; clone ID: LD37010). The entire *abi* open reading frame (ORF) was PCR-amplified and cloned into the pGEM-T easy vector (Promega). W452K, 4YE (Y148E + Y155E + Y248E + Y285E), and 4YF (Y148F + Y155F + Y248F + Y285F) mutations were introduced into *pGEM*-*T*-*abi* via two-step PCR-based mutagenesis. For the transgenic rescue experiments, *abi*, *abi*^*W452K*^, *abi*^*4YE*^, and *abi*^*4YF*^ cDNA inserts were subcloned into pUAST-HA^11^; named *UAS*-*HA*-*abi*, *UAS*-*HA*-*abi*^*W452K*^, *UAS*-*HA*-*abi*^*4YE*^, and *UAS*-*HA*-*abi*^*4YF*^, respectively. We generated *UAS*-*HA*-*abi*^*Δ30–65*^ and *UAS*-*HA*-*abi*^*Δ123–175*^ by sequentially subcloning two cDNA fragments encoding N- and C-terminal Abi relative to the deleted region into pUAST-HA. For expression in *Drosophila* BG2-c2 cells, *HA*-*abi*, *HA*-*abi*^*Δ30–65*^, *HA*-*abi*^*Δ123–175*^ *HA*-*abi*^*W452K*^, *HA*-*abi*^*4YE*^, and *HA*-*abi*^*4YF*^ cDNA inserts were ligated into the pAc5.1 vector (Invitrogen). For expression in *Escherichia* *coli*, cDNA fragments encoding Abi-N (amino acids 1–387), Abi-NΔ30–65, Abi-NΔ123–175, Abi-SH3 (amino acids 388–473), Abi-SH3-W452K, and Abi-C (amino acids 253–473) were subcloned into the pGEX6P1 vector (GE Healthcare). For *abi*-*GAL4*, a 2785-bp fragment of the *abi* 5′ region (−2801 to −17 relative to the translation start site) was PCR-amplified from the genomic clone BACR10E03 (BACPAC Resources) and inserted into the pCaSpeR4 vector (Addgene). Subsequently, GAL4 cDNA amplified from the pGaTB vector^63^ by PCR was inserted into the 3′ end of the *abi* promoter.

For expression in animals or BG2-c2 cells, cDNAs were subcloned into pUAST or pAc5.1, respectively. Full-length cDNAs for *tkv* and *wit* were obtained from DGRC (clone ID: LD45557 and GH13548, respectively). Two-step PCR was performed to generate cDNAs encoding Tkv or Wit with an N-terminal Myc (EQKLISEEDL) or Flag (DYKDDDDK) tag, respectively, after the signal sequence. The *Myc*-*tkv*, *Myc*-*wit*, and *Flag*-*wit* cDNAs were ligated into the pGEM-T easy vector. The *Myc*-*tkv* and *Flag*-*wit* cDNAs moved into pUAST, named *UAS*-*Myc*-*tkv* and *UAS*-*Flag*-*wit*, respectively. The *Myc*-*tkv* and *Myc*-*wit* cDNA inserts were also subcloned into pAc5.1 to create *pAc*-*Myc*-*tkv* and *pAc*-*Myc*-*wit*. The full-length *Rabankyrin* ORF was PCR-amplified from the cDNA RE06111 cDNA template (DGRC) and ligated into the pEGFP-C1 vector (Clontech). The *GFP*-*Rabankyrin* cDNA insert was ligated into pUAST and pAc5.1 to generate *UAS*-*GFP*-*Rabankyrin* and *pAc*-*GFP*-*Rabankyrin*, respectively. The full-length *CtBP* ORF was PCR-amplified from the cDNA GH20987 cDNA template (DGRC) and ligated into the pEGFP-N3 vector (Clontech). The *CtBP*-*GFP* cDNA insert was ligated into pUAST to generate *UAS*-*CtBP*-*GFP*. The coding sequence of the PLCδ_1_ PH domain (amino acids 1–170) was amplified by reverse transcription-PCR from total RNA extracted from human K562 leukemia cells and then subcloned into pAc-EGFP and pAc-mCherry vector to create *pAc*-*PLC*-*PH*-*GFP* and *pAc*-*PLC*-*PH*-*mCherry*, respectively. Wild-type *Abl* and *Abl*^*K417N*^ cDNAs were amplified by reverse transcription-PCR of RNA extracted from *Drosophila* S2R+ cells and PCR from the genomic DNA of *UAS*-*Abl*^*K417N*^ transgenic flies, respectively. The resulting PCR products were subcloned into pAc5.1 containing a C-terminal Myc tag to create *pAc*-*Abl*-*Myc* and *pAc*-*Abl*^*K417N*^-*Myc*. For *pAc*-*kette*-*Myc*, cDNA encoding Kette with a C-terminal Myc tag was amplified from the LD43495 cDNA template (DGRC) by PCR and ligated into the pAc vector. For *pAc*-*GFP*-*Rab5 and pAc*-*GFP*-*Rab7*, the *Rab5* and *Rab7* cDNAs were amplified by reverse transcription-PCR of RNA extracted from *Drosophila* S2R+ cells and inserted into pAc5.1 containing an N-terminal GFP tag. All primers used for cloning are listed in Supplementary Table 2.

For expression of Gbb and HA-Gbb in S2R+ cells, the full-length *gbb* cDNA was PCR-amplified from the genomic DNA of *UAS*-*gbb* transgenic flies (gift from Kristi Warton; Brown University) and inserted into the pGEM-T easy vector. The resulting plasmid was used for two-step PCR to introduce a single HA tag (GGYPYDVPDYAGG) between Thr351 and Arg352 (ref. ^65^). The *gbb* and *HA*-*gbb* cDNA inserts were moved into pAc5.1 to generate *pAc*-*gbb* and *pAc*-*HA*-*Gbb*.

For RNAi experiments in BG2-c2 cells, *abi*, *Abl*, *Rac1*, *SCAR*, *kette*, *Rabankyrin*, *Graf*, and *Chc* dsRNAs were synthesized by in vitro transcription of their cognate DNA templates. To generate DNA templates, we used primers containing the T7 promoter sequence upstream of the following: *abi*, 5′-GCCTCGCATCGATATTCTA-3′ and 5′-ACCATATAGAGCGTATGTG-3′; *Abl*, 5′-GGATCCGGATCGGGGCTGAGC-3′ and 5′-CTCTGAGATGCGGTAGTGATA-3′; *Rac1*, 5′-CAGGCGATCAAGTGCGTCG-3′ and 5′-GAGCAGGGCGCACTTGCGC-3′; *SCAR*, 5′-GTGTATCAGCAGGATGAGC-3′ and 5′-CGCCGTGCACCAGTGCACG-3′; *kette*, 5′-ACCTGGTACAGTGAGGTTC-3′ and 5′-CATCAGTAGACAGGCAGTG-3′; *Rabankyrin*, 5′-GCCAAATCTAGTTAAGAAG-3′ and 5′-GCAGCGGAGATGCCTTATC-3′; *Graf*, 5′-GTTACTCAATCGGGCTCAA-3′ and 5′-AATGGTGCGGCTTCAAATG-3′; and *Chc*, 5′-GCCTGCTGGAAATGAAT-3′ and 5′-CGCTCCACCTCCTTAAT-3′. DNA templates were transcribed with the Megascript T7 Transcription Kit (Ambion) to generate dsRNAs.

To characterize *abi* deletions, the following primers were used for genomic PCR: 5′-GCGGCCGCTTAGACACAAGGCTCTACGTAG-3′ and 5′-CTTCAAGCTCCAGTACTTTGCAGCAC-3′. To analyze the effects of the *abi*^*5*^ mutation on the expression of *abi*, *twinfilin* (*twf*), and *rp49* (control), total RNAs from *abi*^*5*^/*abi*^*5*^ and *abi*^*5*^/*Df* third instar larvae were reverse transcribed. The resulting cDNAs were analyzed by PCR using the following primers: *abi*, 5′-ATGTTGACCGAAACCCCCATG-3′ and 5′-CACGCCAATCTCTCTCCTG-3′; *twf*, 5′-GTCTCACCAAACGGGTATC-3′ and 5′-CGAAATCCTGCTTGTGCCG-3′; and *rp49*, 5′-CACCAGTCGGATCGATATGC-3′ and 5′-CACGTTGTGCACCAGGAACT-3′. To assess the effects of Abi, Abl, and Rac1 loss of function on *dFmr1* RNA expression, total RNA prepared from the larval central nervous system (brain and VNC) was reverse-transcribed into cDNA. The resulting cDNAs were used as templates for real-time PCR with Applied Biosystems SYBR Green PCR Master Mix and the following *dfmr1* primers: 5′-GGATCAGAACATACCACGTG-3′ and 5′-CGCCTCCACGATAGCTGCCAG-3′. The comparative CT method was used to analyze the level of *dFmr1* RNA expression.

### Cell transfection and production of Gbb-conditioned medium

*Drosophila* BG2-c2 neuronal cells (DGRC; Supplementary Table 1) were maintained at 25 °C in Shields and Sang M3 Insect Medium (Sigma-Aldrich) supplemented with 10% heat-inactivated (30 min, 55 °C) fetal bovine serum (FBS; Gibco) and 10 μg/ml insulin (Sigma-Aldrich). *Drosophila* S2R+ cells (DGRC; Supplementary Table 1) were maintained at 25 °C in Schneider’s medium (Gibco) supplemented with 10% FBS. Cells were transfected in serum-free medium using Cellfectin (Invitrogen), according to the manufacturer’s instructions. UAS constructs were cotransfected with *Actin 5C*-*GAL4*. For production of Gbb-conditioned medium, S2R+ cells transfected with *pAc*-*gbb* were incubated in serum-free Schneider’s medium (Gibco) for 120 h. Multiple Tag Fusion (GenScript) was used as protein standard to determine HA-Gbb concentration by western blot using rabbit anti-HA (1:1000; Cell Signaling). Conditioned medium with known HA-Gbb concentration was used as a standard for determining Gbb concentration by western blot using mouse anti-Gbb (GBB 3D6-24; 1:500; DSHB).

### Abi antibody generation

A GST-Abi-C (amino acids 253–473) fusion protein expressed in *E*. *coli* BL21 (Stratagene) was purified with glutathione Sepharose 4B (GE Healthcare). Rats were immunized with purified GST-Abi-C protein (AbFrontier, Korea).

### Western blotting, GST pull-down, and Tkv degradation assay

For western blot analysis of *abi* mutants and transgenes, third instars were homogenized in sodium dodecyl sulfate (SDS) sample buffer (62.5 mM Tris-HCl, pH 6.8, 10% glycerol, 2% SDS, 2.88 mM β-mercaptoethanol, and 0.02% bromophenol blue), boiled for 10 min, and centrifuged at 13,000 × *g* for 15 min. The supernatants were subjected to SDS–polyacrylamide gel electrophoresis and transferred to nitrocellulose membrane (Whatman). Blots were blocked in 5% bovine serum albumin (BSA)/TBST (TBS, 0.1% Tween-20) and incubated with rat anti-Abi (1:500), rabbit anti-HA (1:1000, Cell Signaling Technology), or rabbit anti-β-actin (1:1000; Sigma-Aldrich) overnight at 4 °C. After several washes in TBST, blots were incubated with HRP-conjugated secondary antibodies (1:5000; Jackson ImmunoResearch Laboratories) for 1 h at room temperature. Protein bands were visualized by enhanced chemiluminescence reagents (Thermo Scientific). Full-length western blots are shown in Supplementary Fig. 10.

GST pull-down assays were performed to characterize physical interactions of mutated Abi proteins with SCAR, Kette, or WASp. GST and GST fusion proteins of various Abi regions were expressed in *E*. *coli* BL21 and purified using glutathione-Sepharose 4B (GE Healthcare). S2R+ cells mock-transfected or transfected with *pAc*-*kette*-*Myc* were homogenized in lysis buffer (25 mM Tris-HCl, pH 7.5, 150 mM NaCl, 0.5% Triton X-100, and protease inhibitors) and then centrifuged at 12,000 × *g* for 15 min at 4 °C. Cytoplasmic supernatants were mixed with 10 μg of GST fusion proteins immobilized on glutathione-Sepharose 4B beads for 4 h at 4 °C. Proteins were eluted from the beads in SDS sample buffer for western blot analysis using rabbit anti-Myc (1:1000; Cell Signaling Technology), mouse anti-SCAR (P1C1-SCAR; 1:100; Developmental Studies Hybridoma Bank [DSHB]), or mouse anti-WASp (P5E1-Wasp; 1:100; DSHB).

To assess ligand-induced Tkv degradation, BG2-c2 cells transfected with *pAc*-*Myc*-*tkv* in the presence or absence of *abi* or *Rabankyrin dsRNA* were incubated in serum-free Shields and Sang M3 medium for 3 h. Cycloheximide was added to the culture medium at a final concentration of 50 μg/ml, and cells were further incubated for 3 h. Cells were then stimulated with S2R+-conditioned medium with or without 50 ng/ml Gbb for the indicated times and homogenized in lysis buffer (25 mM Tris-HCl, pH 7.4, 150 mM NaCl, 1% Triton X-100, and protease inhibitors) for western blot analysis using rabbit anti-Myc (1:1000; Cell Signaling) and rabbit anti-β-actin (1:1000; Sigma-Aldrich).

### Immunohistochemistry and imaging of larval tissues

Wandering third instar larvae were dissected in Ca^2+^-free HL3 saline^66^ and fixed in 4% formaldehyde/phosphate-buffered saline (PBS) for 30 min. Fixed larval fillets were then washed in permeabilizing PBST-0.1 (PBS, 0.1% Triton X-100) or detergent-free PBS (for extracellular Gbb labeling) and incubated with primary antibodies in 0.2% BSA/PBST-0.1 or 0.2% BSA/PBS (for extracellular Gbb labeling) at 4 °C overnight. The following antibodies were used: rabbit anti-Gbb^56^ at 1:100; rat anti-Abi at 1:100, fluorescein isothiocyanate (FITC)-conjugated goat anti-HRP and Cy5-conjugated goat anti-HRP (Jackson ImmunoResearch Laboratories) at 1:200, rabbit anti-P-Mad (PS1)^67^ at 1:100, rabbit anti-GluRIIC (gift from Aaron DiAntonio, Washington University) at 1:2000, rabbit anti-HA (Cell Signaling Technology) at 1:100, rabbit anti-Myc (Cell Signaling Technology) at 1:100, mouse anti-Myc (BD Pharmingen) at 1:100, mouse anti-Flag (Sigma-Aldrich) at 1:100, mouse anti-Futsch (22C10; DSHB) at 1:50, mouse anti-Dlg (4F3; DSHB) at 1:500, and mouse anti-Bruchpilot (nc82; DSHB) at 1:10. FITC- and Cy3-conjugated secondary antibodies (Jackson ImmunoResearch Laboratories) were used at 1:200. All antibodies used in this study are listed in Supplementary Table 3.

Images of whole anti-HRP-labeled NMJs were acquired on a FV300 laser-scanning confocal microscope (Olympus) using a Plan Apo ×40 0.9 objective lens. Other fluorescent images were acquired on an LSM 800 confocal microscope (Carl Zeiss) with a C-Apo ×40 1.2W or Plan-Apo ×63 1.25 Oil objective lens. All confocal images were obtained from NMJ 6/7 and NMJ 4 in segment A2. For quantification of NMJ morphology, maximum projection images of NMJ 6/7 were produced from stacked confocal images (1-μm thickness), and total and satellite bouton numbers were counted. A satellite bouton was defined as a single bouton that was not included in a chain of boutons. For quantification of P-Mad, Futsch, Myc-Tkv, Flag-Wit, and extracellular synaptic Gbb levels, images of middle optical sections of individual boutons were acquired and their fluorescence intensities were normalized to that of HRP.

VNC was dissected out from wandering third instar larvae in ice-cold PBS and fixed in 4% formaldehyde/PBS for 30 min at room temperature. Fixed VNCs were washed in PBST-0.3 (PBS, 0.3% Triton X-100) and incubated with primary antibodies in 0.2% BSA/PBST-0.3 for 48 h at 4 °C. The following primary antibodies were used: rabbit anti-P-Smad3 (Epitomics) at 1:200, rabbit anti-HA (Cell Signaling Technology) at 1:100, and mouse anti-Even Skipped (2B8; DSHB) at 1:10. Stained VNCs were collected on a LSM 800 confocal microscope with a Plan Apo ×20 0.8 or Plan Apo ×40 1.3W objective lens.

### Immunofluorescence of BG2-c2 cells

BG2-c2 cells were fixed in 4% formaldehyde/PBS for 10 min and permeabilized with PBST-0.2 (PBS, 0.2% Triton X-100) or saponin/PBS-0.1 (PBS, 0.1% saponin; for TMR-Dex-labeled cells only) for 10 min, blocked with 1% BSA/PBS 30 min, and incubated with primary antibodies in 1% BSA/PBS for 1 h at room temperature. The following antibodies were used: rat anti-Abi at 1:100, mouse anti-HA (BioLegend) at 1:100, and rabbit anti-Myc (Cell Signaling Technology) at 1:100. FITC- and Cy5-conjugated secondary antibodies (Jackson ImmunoResearch Laboratories) were used at 1:200. Actin filaments were stained with 6.6 μM rhodamine-phalloidin (Molecular Probes). For each cell, single confocal slice or *Z* stack of optical sections (0.33 μm) were taken with an LSM 800 confocal microscope using a Plan Apo ×63 1.4 Oil objective.

### Dextran uptake assay

BG2-c2 cells were subjected to serum deprivation for 6 h. Starved cells were pulsed with 2 mg/ml of 70 kDa TMR-Dex (Molecular Probes) in the S2R+-conditioned medium containing 50 ng/ml Gbb for 5 min. In some experiments, mock-transfected cells were pretreated with DMSO, 25 μM LY294002 (Sigma-Aldrich), or 100 μM EIPA (Sigma-Aldrich) over the last 30 min of the serum starvation step. TMR-Dex-pulsed cells were washed three times with ice-cold PBS, fixed in 4% formaldehyde/PBS for 10 min, washed three times with PBS, and stained with 1 μg/ml 4,6-diamidino-2-phenylindole (Molecular Probes) in PBS. Larval NMJ preparations were also pulsed with 70 kDa TMR-Dex as described above. After being washed with ice-cold PBS, the samples were fixed in 4% formaldehyde/PBS for 30 min and further processed for FITC-HRP labeling to visualize NMJ structure. A *Z* stack of optical sections (0.33-μm thick) was taken with a LSM 800 confocal microscope using a Plan Apo ×63 1.4 Oil objective. Maximum-intensity projection images were used to measure the number of TMR-Dex-positive puncta (>0.2 μm in diameter) per cell or three terminal boutons at each NMJ branch in the ImageJ software (NIH).

### BMPR internalization and trafficking studies

For the BMPR internalization assay, BG2-c2 cells transfected with *pAc*-*Myc*-*tkv* or *pAc*-*Myc*-*wit* alone or in combination with *abi*, *Rabankyrin*, *Chc*, or *Graf* dsRNA were serum-starved for 6 h and incubated at 4 °C with 2.5 μg/ml mouse anti-Myc antibody (BD Pharmingen) in serum-free medium for 30 min to label surface Myc-Tkv or Myc-Wit BMPRs. In some experiments, cells were pretreated with DMSO, 25 μM LY294002, 100 μM EIPA, or 20 μM Dynasore (Sigma-Aldrich) over the last 30 min of the serum starvation step. Cells were incubated at 25 °C in conditioned medium containing 0 or 50 ng/ml Gbb for 5 min to allow internalization. Cells were then fixed in 4% formaldehyde/PBS for 10 min and incubated with FITC-conjugated anti-mouse secondary antibody to label surface BMPRs. Cells were then washed in PBS, permeabilized with 0.2% Triton X-100/PBS for 10 min, and incubated with Cy3-conjugated anti-mouse secondary antibody. A *Z* stack of optical sections (0.35-μm thick) was taken with an LSM 800 laser-scanning confocal microscope using a Plan Apo ×63 1.4 Oil objective. For quantification of internalization, we used ImageJ to measure surface and internal Myc-Tkv or Myc-Wit fluorescence intensities as the integrated pixel intensities in the green and red channels, respectively. The ratio of mean internalized to surface fluorescence intensities was used as the internalization index.

For BMPR trafficking studies, live BG2-c2 cells transfected with *pAc*-*Myc*-*tkv* or *pAc*-*Myc*-*wit* alone or in combination with *abi*, *Rabankyrin*, *Chc*, or *Graf* dsRNA were prelabeled with anti-Myc antibody as described above. Cells were then pulsed at 25 °C for 2 min with 2 mg/ml TMR-Dex in S2R+-conditioned medium containing 0 or 50 ng/ml Gbb and chased at 25 °C for 3 or 18 min in serum-free Schneider’s medium lacking Gbb. In some experiments, cells were additionally treated with 1 μM Lysotracker (Molecular Probes) during the last 5 min of the chase to label lysosomes. Cells were then fixed in 4% formaldehyde/PBS for 10 min, permeabilized with 0.1% saponin/PBS for 10 min, and incubated with a FITC-conjugated secondary antibody in 1% BSA/PBS for 1 h. Fluorescence images were obtained with a Plan Apo ×63 1.4 Oil objective on an LSM 800 laser-scanning confocal microscope. The index of colocalization between Myc-Tkv/Myc-Wit and Lysotracker or TMR-Dex was manually counted using ImageJ.

### NMJ electrophysiology and FM1-43FX dye uptake assay

TEVC electrophysiology was performed on the wandering third instar NMJ. Staged larvae were glued on sylgard-coated coverslip and dissected longitudinally along the dorsal midline. Peripheral motor nerves were cut at the base of the VNC. Dissections and stimulations were done at 18 °C in 5 mM HEPES, pH 7.2, 128 mM NaCl, 2 mM KCl, 4 mM MgCl_2_, 1 mM CaCl_2_, 70 mM sucrose, and 5 mM trehalose. Preparations were viewed with a Zeiss Axioskop microscope using a ×40 water immersion objective. The motor nerve bundle of hemisegment A2 or A3 was sucked into a fire-polished suction electrode. Muscle 6 in segments A2/A3 was impaled with two intracellular microelectrodes (1-mm outer diameter borosilicate capillaries; World Precision Instruments) of ~20 MΩ resistance filled with 3 M KCl. Muscles were clamped at −60 mV using an Axoclamp-2B amplifier. Motor nerves were stimulated using 0.5 ms suprathreshold voltage stimuli at 0.2 Hz from a Grass S88 stimulator. Nerve stimulation-evoked EJC recordings were filtered at 2 kHz. To quantify EJCs, 10 consecutive traces were averaged and the average peak value recorded. Clampex 10.6 was used for data acquisition, Clampfit 10.6 for data analysis, GraphPad Prism 7 for statistical analysis, and Adobe Photoshop Elements for data presentation.

For FM1-43FX dye uptake assay, wandering third instar larvae were dissected on sylgard plates in Ca^2+^-free HL3 saline. Preparations were incubated in HL3 saline containing 90 mM KCl, 5 mM CaCl_2_, and 4 μM FM1-43FX for 2 min. The samples were washed in Ca^2+^-free HL3 saline for 10 min and then fixed in 4% formaldehyde in PBS for 30 min. Images of synaptic boutons at NMJ 6/7 in segment A2 were obtained with a Plan Apo ×63 1.4 Oil objective on a Zeiss LSM 800 laser-scanning confocal microscope. The fluorescence intensity was measured using the ZEN imaging software (Carl Zeiss).

### Electron microscopy

Wandering third instars overexpressing presynaptic BMPR Tkv (*C155*-*GAL4*/+; *UAS*-*Myc*-*tkv*/+) were dissected in Ca^2+^-free HL3 saline, treated with 50 ng/ml Gbb for 5 min at 25 °C, and rinsed with 0.2 M sodium cacodylate buffer (pH 7.2). Preparations were fixed overnight at 4 °C in 0.2 M sodium cacodylate buffer (pH 7.2) containing 2.5% glutaraldehyde. Secondary fixation was done for 1 h in 0.1 M sodium cacodylate containing 1% osmium tetroxide. En bloc uranyl acetate staining was performed using 2% uranyl acetate, followed by ethanol dehydration, propylene oxide infiltration, and resin embedding. Type Ib boutons at NMJ 6/7 (segments A2 and A3) were serially sectioned (60 nm) on a Philips FEI T-12 transmission electron microscope operating at 100 kV, with images collected using a 2k-by-2k Gatan CCD camera. Macropinosome diameters were measured in Image J.

### Live cell imaging

BG2-c2 cells transfected with *pAc*-*GFP*-*abi* and *pAc*-*PLC*-*PH*-*mCherry* were transferred to a culture dish (SPL Life Sciences) and incubated with serum-free Shields and Sang M3 Insect Medium (Sigma-Aldrich) for 6 h. Culture medium was replaced with control or Gbb-conditioned (50 ng/ml Gbb) medium. Time-lapse images were collected at 1 stack/1.86 s for 10 min with an LSM 800 laser-scanning confocal microscope using a Plan Apo ×100 1.4 Oil objective.

### Statistical analysis

Data are presented mean values ± s.e.m of at least three experiments. Statistical significance was determined by Student’s *t* test or one-way analysis of variance followed by post hoc pairwise comparisons of means using Tukey–Kramer test.

### Reporting summary

Further information on experimental design is available in the Nature Research Reporting Summary linked to this article.

## Supplementary information

Supplementary Information

Reporting Summary

## Data Availability

The data that support the findings of this study are available from the corresponding author upon request.
