# Supplementary material for: BMP-dependent synaptic development requires Abi-Abl-Rac signaling of BMP receptor macropinocytosis
Source: Nat Commun. 2019 Feb 8;10:684. doi: 10.1038/s41467-019-08533-2 (PMC6368546; doi:10.1038/s41467-019-08533-2)
Supplement: Supplementary file 1 — Supplementary Information [file 41467_2019_8533_MOESM1_ESM.pdf]

# Supplementary Information

# BMP-Dependent Synaptic Development Requires Abi-Abl-Rac Signaling of BMP Receptor Macropinocytosis

**Kim et al.**

**Supplementary Note 1. Abi protein interacts with SCAR/WAVE via the WAB domain, Kette via the HHR domain, and WASp via the SH3 domain**

GST pull-down experiments revealed that GST-Abi-NΔ30-65 (deletion of amino acids 30-65 in the WAB domain) and GST-Abi-NΔ123-175 (deletion of amino acids 123-175 in the HHR domain) fail to interact with SCAR and Kette, respectively (Supplementary Fig. 2b). In contrast, Abi lacking the SH3 domain (GST-Abi-N) interacts with both SCAR and Kette (Supplementary Fig. 2b). Pull-down experiments also showed that the point mutation W452K in the Abi SH3 domain (Abi-SH3-W452K) completely abolishes binding interactions with WASp (Supplementary Fig. 2b). An analogous mutation in mammalian cortactin has been also reported to abolish binding to N-WASP[1]. These Abi mutants allowed us to dissect Abi requirements in synaptic regulation.

**Supplementary Note 2. Regulation of dFMRP-Futsch pathway and MT stability by Abi, Abl, and Rac1**

To investigate whether synaptic defects from Abi, Abl, or Rac1 loss requires dFMRP, we tested interactions of Abi, Abl, and Rac1 with dFMRP. First, we found Abi, Abl, and Rac1 all negatively regulate dFMRP expression in the larval central nervous system (Supplementary Fig. 4a). Second, we find transheterozygous interactions between *abi*, *Abl*, or *Rac1* and *dFmr1* at the NMJ, with synaptic overgrowth and supernumerary satellite boutons in transheterozygotes but no single heterozygotes (Supplementary Fig. 4b). Third, the neuronal overexpression of dFMRP completely suppresses synaptic overgrowth at $abi^{5}/Df$, $Abl^{1}/Abl^{4}$, and *C155-GAL4*/+; $UAS\text{-}Rac1^{T17N}$/+ NMJs (Supplementary Fig. 4c). Importantly, synaptic development is not different between animals overexpressing dFMRP alone versus $abi^{5}/Df$, $Abl^{1}/Abl^{4}$, or *C155-GAL4*/+; $UAS\text{-}Rac1^{T17N}$/+ larvae overexpressing dFMRP. Together, these

results suggest that Abi, Abl, and Rac1 regulate synaptic development via modulation of the BMP-dFMRP pathway.

We next tested effects of *abi*, *Abl*, or *Rac1* loss-of-function on synaptic MT stability. Futsch/MAP1B levels are significantly increased in NMJ axons and presynaptic boutons of $abi^{5}/Df$ and $Abl^{1}/Abl^{4}$ mutants, as well as of wildtype animals expressing Rac1-T17N, $SCAR^{RNAi}$, or $kette^{RNAi}$ (Supplementary Fig. 4d, e). We find a significant increase of synaptic boutons with microtubule loops, indicating that loss of *abi*, *Abl*, *Rac1*, and components of the SCAR complex enhances MT stability at the NMJ (Supplementary Fig. 4d). To ascertain the involvement of increased synaptic MT stability, we examined the effect of the MT-severing drug vinblastine on the synaptic overgrowth induced by mutations in *abi* or *Abl*, as well as by neuronal expression of Rac1-T17N, $SCAR^{RNAi}$, or $kette^{RNAi}$. Vinblastine administration (1 μM) does not alter synaptic structure in genetic controls but suppresses synaptic overgrowth in all mutants, indicating that MT dysregulation is responsible for synaptic defects in *abi*, *Abl*, *Rac1*, *SCAR*, and *kette* loss-of-function mutants (Supplementary Fig. 4f). Taken together, these results suggest that the Abi-SCAR complex acts downstream of Abl and Rac1 to regulate synaptic development via the BMP-dFMRP-Futsch-MT stability pathway in the presynaptic terminal.

**Supplementary Note 3. Gbb induces Abi-dependent macropinocytosis in BG2-c2 cells**

To assay macropinocytosis in neuronal cells, serum-starved BG2-c2 cells were stimulated with Gbb-conditioned media (5 to 200 ng/ml Gbb; 5 min) in the presence of TMR-Dex (70 kDa). BG2-c2 cells incubated without Gbb display low levels of TMR-Dex uptake (Supplementary Fig. 5a, b), but Gbb stimulation induces TMR-Dex uptake in a dose-dependent manner (maximal at 50 ng/ml). Uptake is strongly impaired by two

macropinocytosis inhibitors (LY294002 and ethylisopropylamiloride (EIPA)), or by knockdown of macropinocytosis regulator Rabankyrin (Supplementary Fig. 5c-g). Moreover, Gbb stimulation also potently induces formation of F-actin rich membrane ruffles (Supplementary Fig. 5h), which are functionally coupled to macropinocytosis[2]. Gbb-induced TMR-Dex uptake and membrane ruffling are both abolished by *abi* dsRNA (Supplementary Fig. 5e-h). Knockdown of Abl, Rac1, SCAR, or Kette also impairs Gbb-induced TMR-Dex uptake into BG2-c2 cells. These results suggest Gbb induces bona-fide macropinocytosis in BG2-c2 cells dependently of Abi, Abl and Rac1-SCAR signaling.

We then examined the association of Abi with macropinosomes using two approaches. First, we immunostained Gbb-stimulated cells engulfing TMR-Dex with anti-Abi antibody. This experiment clearly reveals that Abi also associates with closed macropinosomes containing TMR-Dex (Supplementary Fig. 5i). Second, we performed a TMR-Dex pulse-chase experiment (Supplementary Fig. 6). In a 2 min pulse, internalized TMR-Dex labeled peripheral, punctate structures colocalized with HA-Abi. Colocalization significantly diminishes after an additional 1-3 min chase, when TMR-Dex-labelled macropinosomes acquire early endosome and macropinosome markers, GFP-Rab5 and GFP-Rabankyrin. After an 18-min chase, with dextran in GFP-Rab7-positive endosomes, TMR-Dex colocalization with HA-Abi is no longer observed. These results demonstrate association of Abi with early macropinosomes.

**Supplementary Note 4. Gbb-induced and Abi-dependent macropinocytosis is not involved in synaptic vesicle endocytosis**

Upon stimulation with depolarizing saline (90 mM $K^+$), NMJs in *abi* mutant larvae or larvae expressing $Rabankyrin^{RNAi}$ or $CtBP^{RNAi}$ in neurons strongly incorporate the fluorescent styryl

dye FM1-43FX (Supplementary Fig. 8h, i). The dye uptake is comparable to the wildtype controls. In a companion experiment, NMJs in temperature-sensitive *dynamin* (*shi*$^{ts1}$) mutants fail to efficiently take up the FM dye at the restrictive temperature (35 $^{o}$C; Supplementary Fig. 8i), confirming that the assay works as expected. These results indicate that Abi-dependent macropinocytosis is not required for efficient synaptic vesicle recycling during depolarizing stimulation and, together with reported dynamin independency of macropinocytosis[3], further suggest that Gbb-induced macropinocytosis is a process distinct from activity-dependent bulk endocytosis (ADBE) at the synapse.

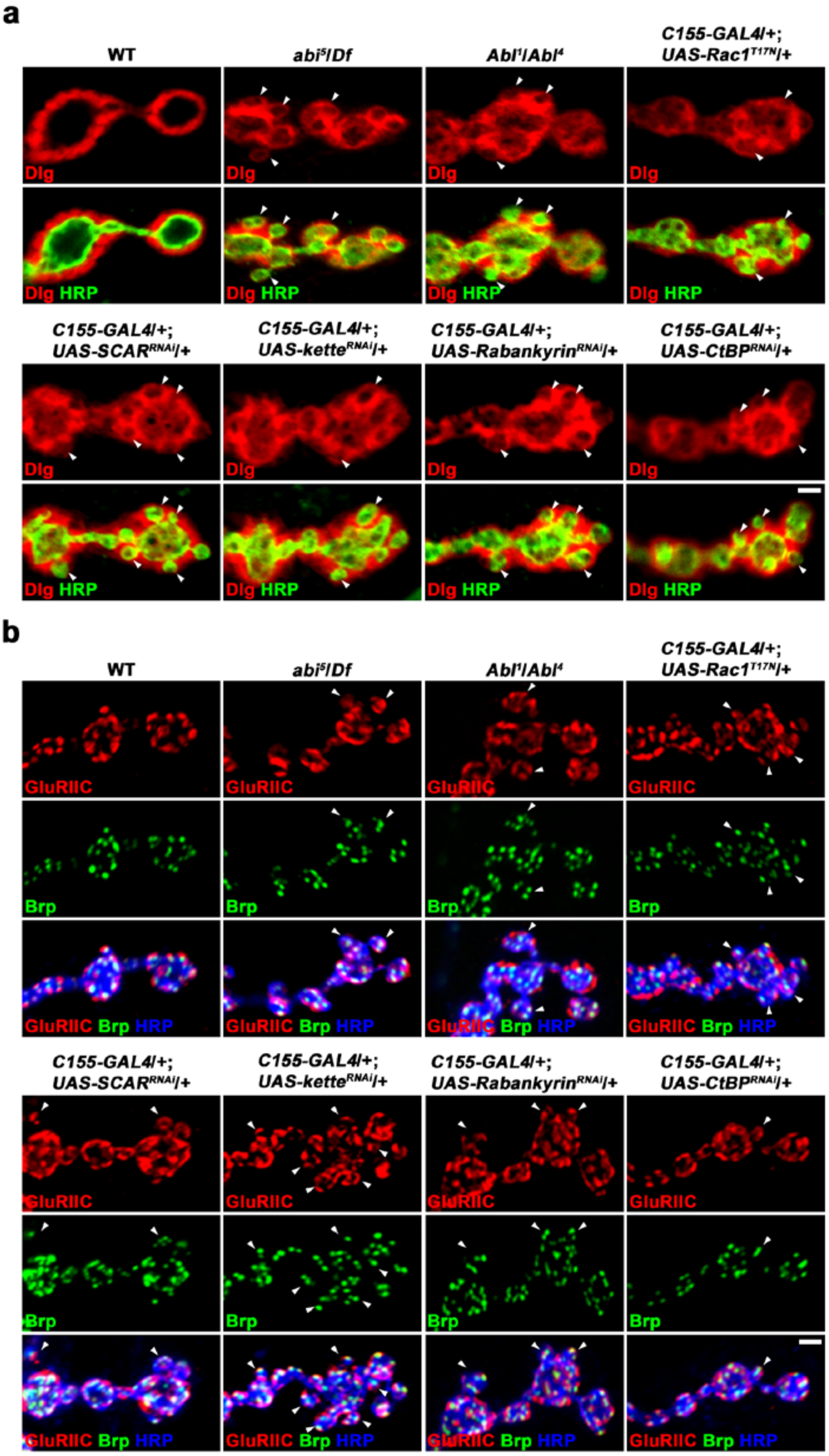


**Supplementary Figure 1.** Characterization of satellite boutons in *Drosophila* mutants

analyzed in this study. Confocal images of NMJ 6/7 in wildtype, *abi*$^{5}$*/Df*, *Abl*$^{1}$/*Abl*$^{4}$, *C155-GAL4*/+; *UAS-Rac1*$^{T17N}$/+, *C155-GAL4*/+; *UAS-SCAR*$^{RNAi}$/+, *C155-GAL4*/+; *UAS-kette*$^{RNAi}$/+, *C155-GAL4*/+; *UAS-Rabankyrin*$^{RNAi}$/+, and *C155-GAL4*/+; *UAS-CtBP*$^{RNAi}$/+ third instar larvae doubly labeled with anti-HRP and anti-Dlg (**a**) or triply labeled with anti-HRP, anti-Brp, and anti-GluRIIC (**b**). Arrowheads indicate satellite boutons. Scale bars: 2 μm.

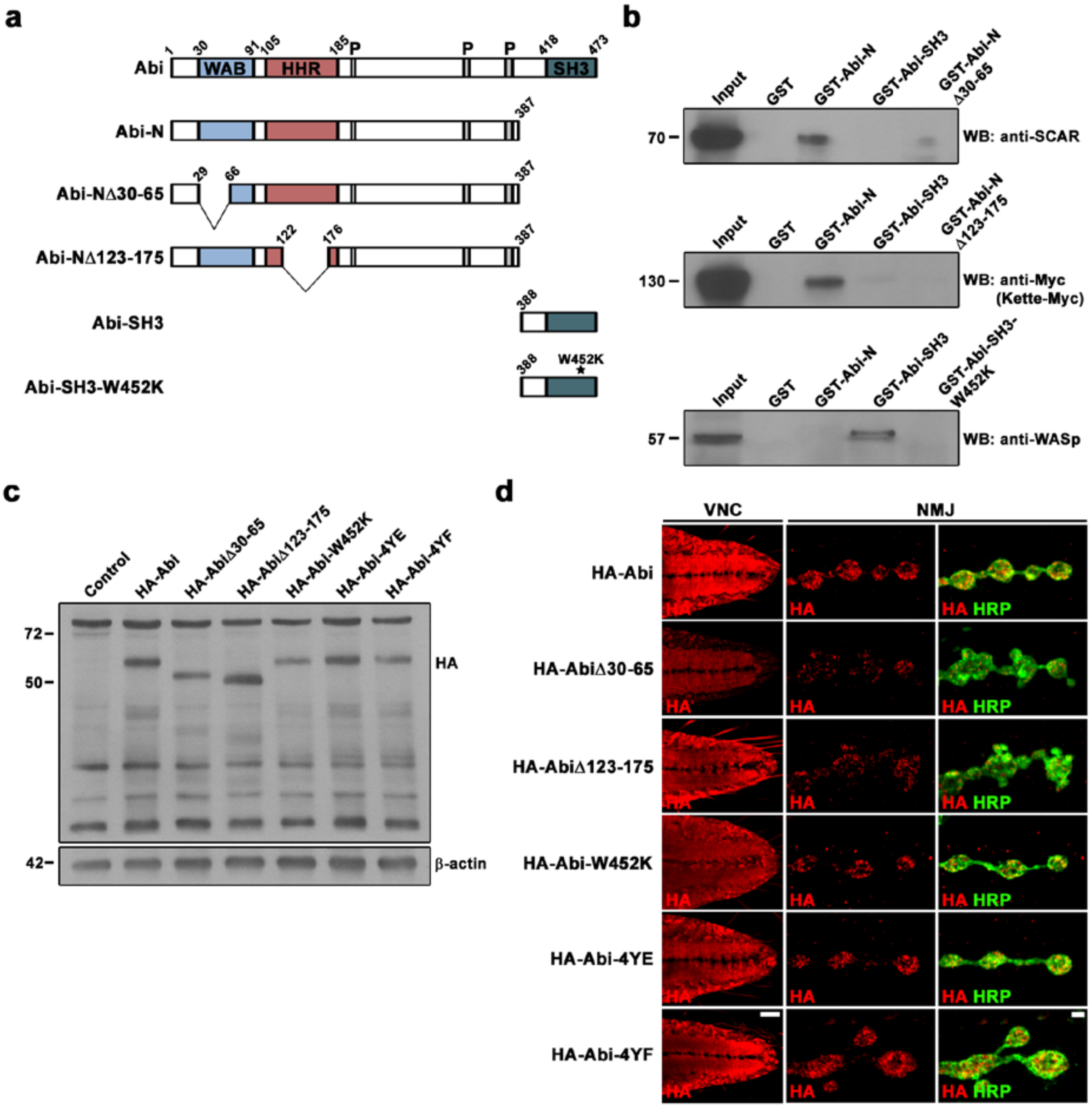


**Supplementary Figure 2.** Analysis of *abi* transgenes used in the rescue experiments. **a** Schematic view of the domain structure of Abi variants used in GST pull-down assays. **b** In GST pull-down assays, lysates from S2R+ cells mock-transfected (top and bottom panels) or transfected with Kette-Myc (middle panel) were incubated with GST alone or Abi proteins (amino-acid boundaries are indicated) fused to GST. Lysates and bound proteins were analyzed by Western blotting using an anti-SCAR, anti-Myc, or anti-WASp antibody. **c**, **d** Analysis of transgenic expression of HA-Abi and its mutants. **c** Western blot analysis of

extracts from *da-GAL4*/+ (control), *da-GAL4*/*UAS-HA-abi* (HA-Abi), *UAS-HA-abi*$^{\Delta 30\text{-}65}$/+; *da-GAL4/*+ (HA-AbiΔ30-65), *da-GAL4/UAS-HA-abi*$^{\Delta 123\text{-}175}$ (HA-AbiΔ123-175), *da-GAL4*/*UAS-HA-abi*$^{W452K}$ (HA-Abi-W452K), *da-GAL4*/*UAS-HA-abi*$^{4YE}$ (HA-Abi-4YE), and *da-GAL4*/*UAS-HA-abi*$^{4YF}$ (HA-Abi-4YF) larvae. The blot was sequentially probed with anti-HA and anti-β-actin antibodies. **d** Single confocal slices of the ventral nerve cord (VNC) and NMJ 6/7 branches of *C155-GAL4/*+; *UAS-HA-abi,Df/abi*$^{5}$ (HA-Abi), *C155-GAL4/*+; *UAS-HA-abi*$^{\Delta 30\text{-}65}$/+; *abi*$^{5}$*/Df* (HA-AbiΔ30-65), *C155-GAL4/*+; *UAS-HA-abi*$^{\Delta 123\text{-}175}$*,Df/abi*$^{5}$ (HA-AbiΔ123-175), *C155-GAL4/*+; *UAS-HA-abi*$^{W452K}$*,Df/abi*$^{5}$ (HA-Abi-W452K), *C155-GAL4/*+; *UAS-HA-abi*$^{4YE}$*,Df/abi*$^{5}$ (HA-Abi-4YE), and *C155-GAL4/*+; *UAS-HA-abi*$^{4YF}$*,Df/abi*$^{5}$ (HA-Abi-4YF). Scale bars: 50 μm (for VNC); 2 μm (for NMJ).

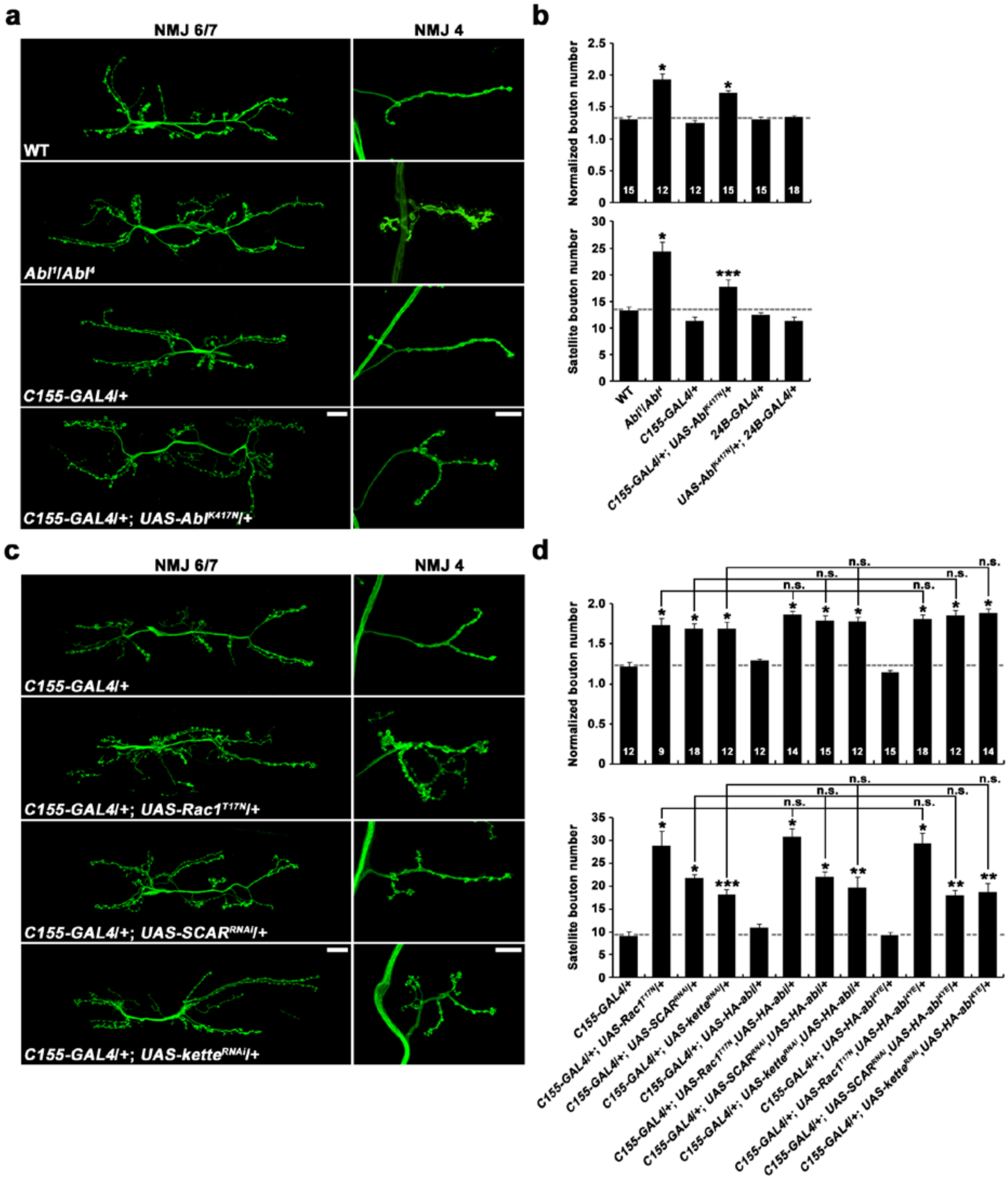


**Supplementary Figure 3.** Loss of presynaptic Abl, Rac1, SCAR, or Kette induces NMJ phenotypes similar to *abi* mutants. **a**, **b** Loss-of-function phenotypes of *Abl*. **a** Confocal images of anti-HRP labeled NMJs in wildtype, *Abl*$^{1}$/*Abl*$^{4}$, *C155-GAL4*/+, *C155-GAL4*/+; *UAS-Abl*$^{K417N}$/+, *24B-GAL4*/+, and *UAS-Abl*$^{K417N}$/+; *24B-GAL4*/+ third instars. **b**

Quantification of overall bouton number normalized to muscle surface area and satellite bouton number at NMJ 6/7 in the indicated genotypes. **c**, **d** Loss-of-function phenotypes of *Rac1*, *SCAR*, and *kette* and their interactions with *abi*. **c**, Confocal images of HRP-labelled NMJ 6/7 (left panels) and NMJ 4 (right panels) in *C155-GAL4*/+, *C155-GAL4*/+; *UAS-Rac1*$^{T17N}$/+, *C155-GAL4*/+; *UAS-SCAR*$^{RNAi}$/+, and *C155-GAL4*/+; *UAS-kette*$^{RNAi}$/+ third instar larvae. **d** Quantification of overall bouton number normalized to muscle surface area and satellite bouton number at NMJ 6/7 in indicated genotypes. Bar graphs indicate mean ± s.e.m. The number of NMJs analyzed for each genotype is indicated inside the bars. Statistical analyses were performed by one-way ANOVA with Tukey-Kramer post hoc test. All comparisons are made against wildtype (**b**) or *C155-GAL4/+* (**d**) unless indicated (*$P$ < 0.001; **$P$ < 0.01; ***$P$ < 0.05; n.s., not significant). Scale bars: 20 μm.

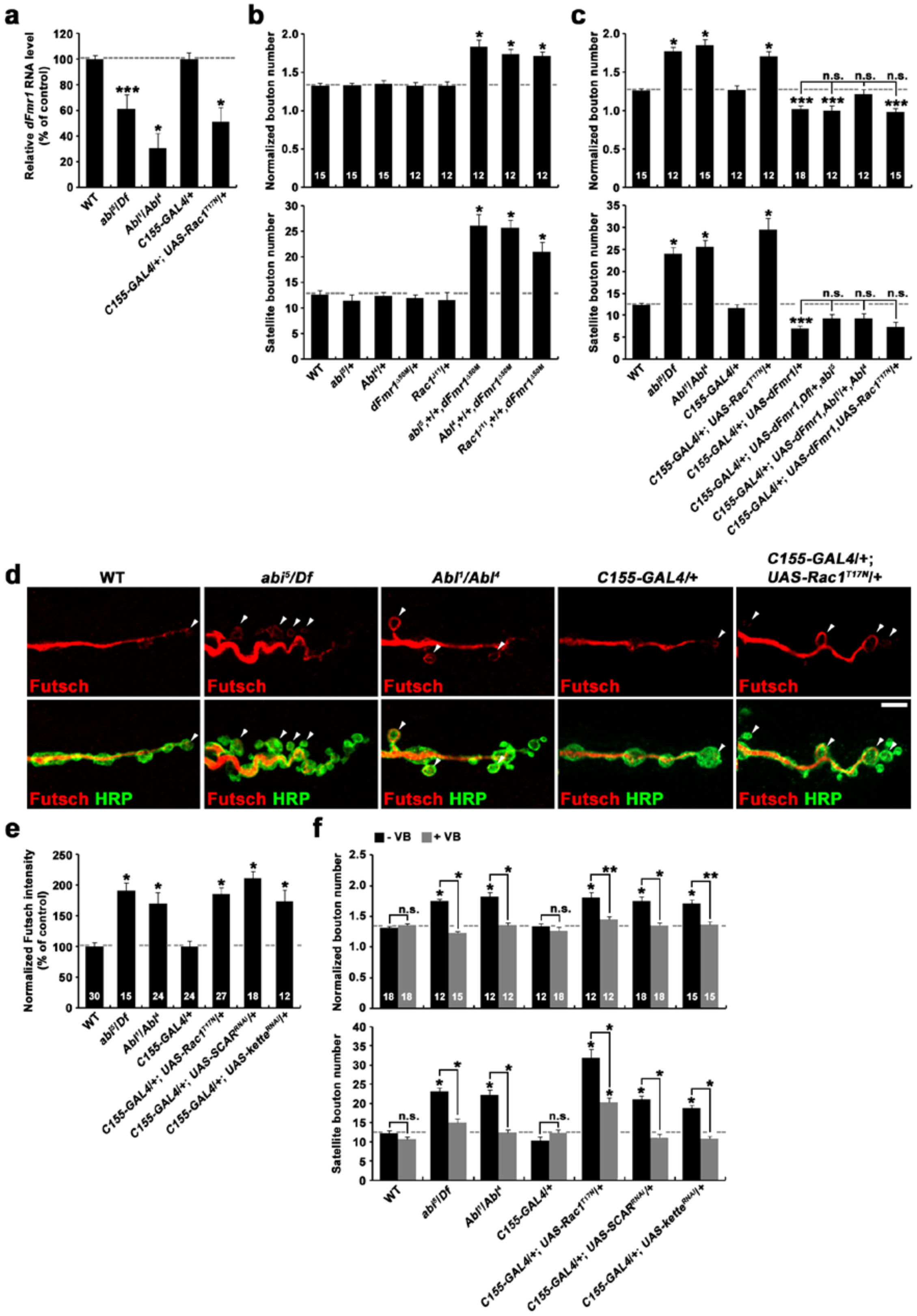


**Supplementary Figure 4.** Abi, Abl, and Rac1 regulate synaptic development by controlling

microtubule stability through the dFMRP-Futsch pathway. **a** Quantification of *dFmr1* mRNA expression in larval CNS (brain and VNC) of the indicated genotypes using quantitative real-time PCR (n = 4, 10 larvae each). *rp49* was used as the internal control. **b** Quantification of synaptic structure at NMJ 6/7 demonstrating transheterozygous interactions between *dFmr1* and *abi*, *Abl*, or *Rac1*. **c** Quantification of synaptic structure at NMJ 6/7 shows that presynaptic expression of *dFmr1* suppresses synaptic overgrowth in *abi* or *Abl* mutant larvae, as well as in larvae coexpressing *UAS-Rac1*$^{T17N}$. **d** Single confocal slice of NMJ 6/7 stained with anti-Futsch and anti-HRP in wildtype, *abi*$^{5}$/*Df*, *Abl*$^{1}$/*Abl*$^{4}$, *C155-GAL4*/+, and *C155-GAL4*/+; *UAS-Rac1*$^{T17N}$/+. Arrowheads indicate presynaptic Futsch-positive loops. Scale bar, 5 μm. **e** Quantification of ratio of mean Futsch to HRP fluorescence intensities in the indicated genotypes. **f** Quantification of synaptic structure at NMJ 6/7 in the indicated genotypes grown in the absence (- VB) or presence (+ VB) of 1 μM vinblastine shows that VB administration suppresses the synaptic overgrowth caused by mutations in *abi* or *Abl*, as well as by neuronal expression of dominant-negative Rac1-T17N, *SCAR* RNAi, or *kette* RNAi. Bar graphs indicate mean ± s.e.m. The number of NMJs analyzed for each genotype is indicated inside the bars. All comparisons are made against wildtype unless indicated (one-way ANOVA with Tukey-Kramer post hoc test; *$P < 0.001$; **$P < 0.01$; ***$P < 0.05$; n.s., not significant).

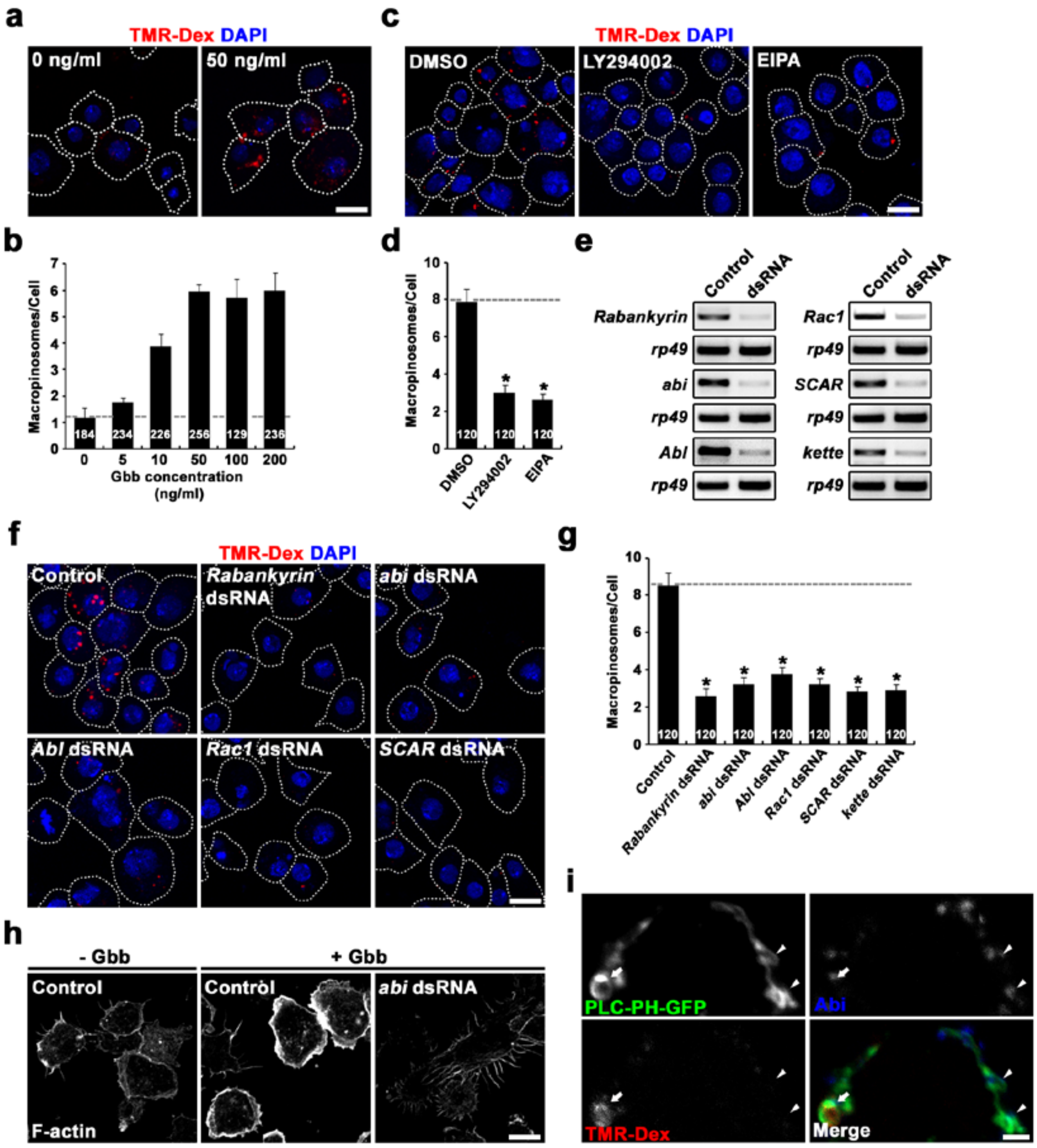


**Supplementary Figure 5.** Abi, Abl, Rac1, Kette, and SCAR are required for Gbb-induced macropinocytosis in BG2-c2 cells. **a** Confocal images of cells stained with DAPI. BG2-c2 cells were serum-starved for 6 hr and incubated for 5 min with TMR-dextran (70 kDa, 2 mg/ml), in the absence or presence of 50 ng/ml Gbb. **b** Quantification of the number of TMR-Dex-filled macropinosomes (puncta > 0.2 μm in diameter) per cell. Cells were treated as in **a** with increasing amounts of Gbb. **c** Effects of the PI3K inhibitor LY294002 and the $Na^+/H^+$ exchanger inhibitor EIPA on Gbb-induced macropinocytosis. Confocal images of starved cells

pretreated with 25 μM LY294002 or 100 μM EIPA for 30 min, prior to the incubation for 5 min with 2 mg/ml TMR-Dex and 50 ng/ml Gbb. **d** Quantification of the number of TMR-Dex-filled macropinosomes per cell. **e** RT-PCR analysis for cells transfected with dsRNA versus the indicated genes. **f** Confocal images of cells depleted for the indicated genes, incubated for 5 min with 2 mg/ml TMR-Dex and 50 ng/ml Gbb, and stained with DAPI. **g** Quantification of the number of TMR-Dex-filled macropinosomes per cell in the indicated genotypes. **h** Single confocal sections of control or Abi-knockdown cells stained for F-actin using rhodamine-phalloidin. Cells were serum-starved for 6 h and incubated for 5 min in the absence (- Gbb) or presence (+ Gbb) of 50 ng/ml Gbb. **i** A single confocal section of a PLC-PH-GFP-expressing cell stained with anti-Abi antibody. Cells were serum-starved for 6 h and incubated with 2 mg/ml TMR-Dex and 50 ng/ml Gbb for 3 min, prior to immunostaining. TMR-Dex-negative unclosed macropinocytic cups are indicated by arrowheads and TMR-Dex-positive closed macropinosomes by arrows. Bar graphs indicate mean ± s.e.m. Statistical analyses were performed by one-way ANOVA with Tukey-Kramer post hoc test. Comparisons are made against DMSO-treated cells (**d**) or mock-treated control cells (**g**). $^*P < 0.001$. Scale bars: **a**, **c**, **f**, **h**, 10 μm; **i**, 1 μm.

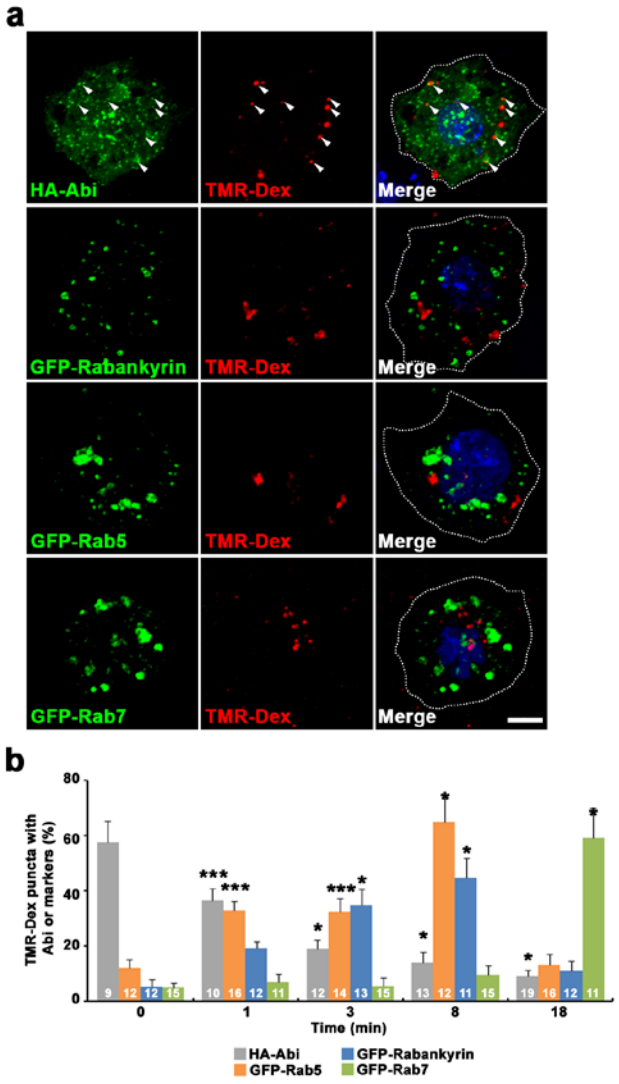


**Supplementary Figure 6.** Abi is associated with Gbb-induced early macropinocytic structures. **a** Confocal images of HA-Abi-, GFP-Rabankyrin-, GFP-Rab5-, or GFP-Rab7-expressing BG2-c2 cells serum-starved for 6 h, pulsed with TMR-Dex (70 kDa, 2 mg/ml) in the presence of 50 ng/ml Gbb for 2 min, and stained with anti-HA antibody. Arrowheads indicate TMR-Dex puncta colocalizing with HA-Abi. **b** HA-Abi-, GFP-Rabankyrin-, GFP-Rab5-, or GFP-Rab7-expressing BG2-c2 cells were pulsed with TMR-Dex as in **a** and chased for the indicated times. Bar graph indicating mean ± s.e.m. from 3 independent experiments show percentage of TMR-Dex-containing macropinosomes associated with each protein. The number of cells analyzed is indicated inside bars. Comparisons are made against cells at 0-

min chase (one-way ANOVA with Tukey-Kramer post hoc test; *$P$ < 0.001; ***$P$ < 0.05). Scale bar: 5 μm.

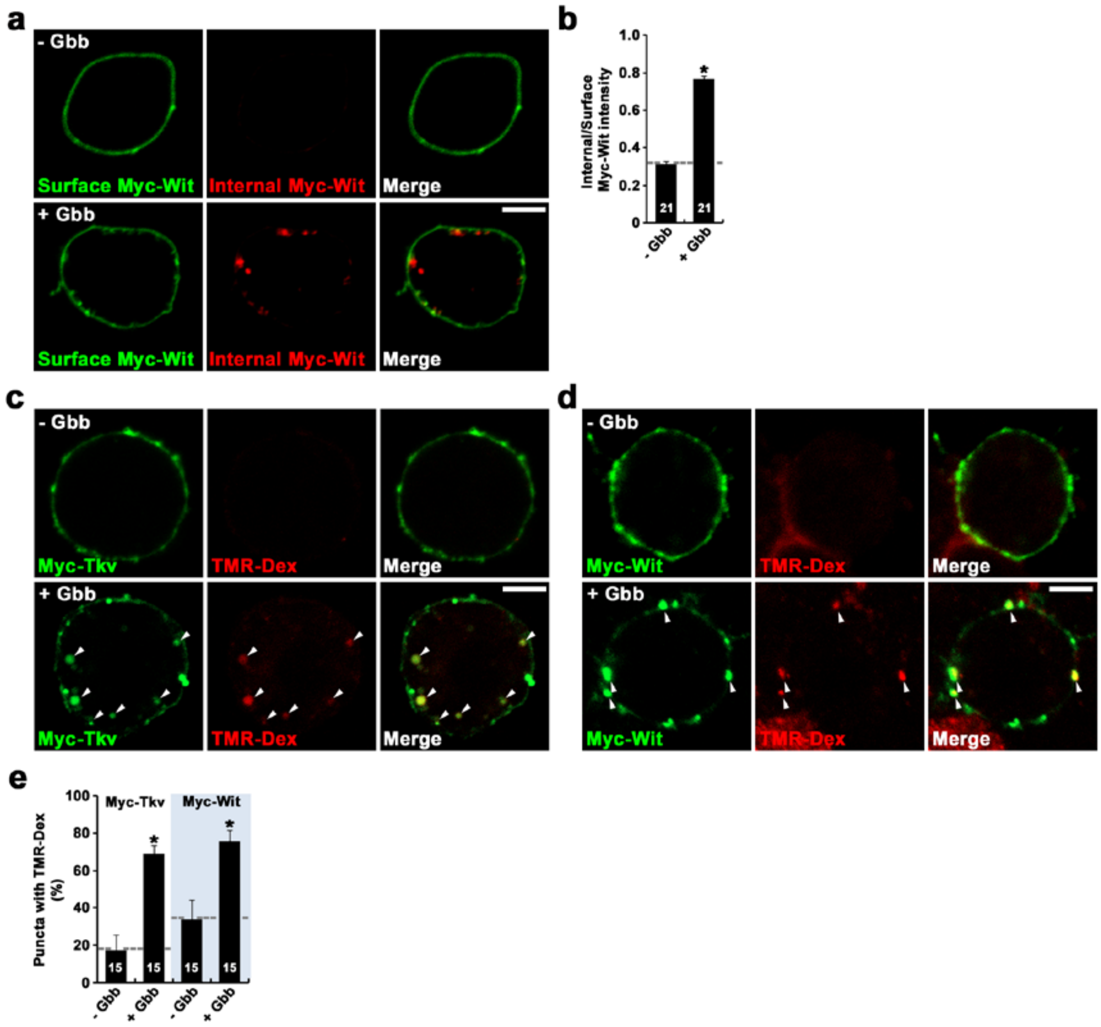


**Supplementary Figure 7.** Gbb-induced macropinocytosis of BMPRs. **a** Single confocal slices through the middle of BG2-c2 cells transfected with Myc-Wit. Transfected live cells were prelabeled with anti-Myc antibody at 4 ºC for 30 min and then incubated in the absence (- Gbb) or presence (+ Gbb) of 50 ng/ml Gbb at 25 °C for 5 min. After fixing of cells, cell surface and internalized receptors were sequentially stained with fluorescent-labeled secondary antibodies under nonpermeant (green) and permeant (red) conditions, respectively. **b** Quantification of internalized-to-surface Myc-Wit ratio. **c**, **d** BG2-c2 cells transfected with Myc-Tkv (**c**) or Myc-Wit (**d**) were prelabeled with anti-Myc antibody at 4 ºC for 30 min. Cells were pulsed with 2 mg/ml TMR-Dex (70 kDa) in the absence (- Gbb) or presence (+

Gbb) of 50 ng/ml Gbb at 25 $^{o}$C for 2 min, chased for 3 min, and stained for anti-Myc. Single confocal sections are shown. **e** Quantification of colocalization between internalized Myc-Tkv/Myc-Wit and TMR-Dex. Bar graphs indicate mean ± s.e.m. The number of cells analyzed for each genotype is indicated inside the bars. All comparisons are made against Gbb-treated control cells unless indicated (Student's *t*-test; *$P < 0.001$). Scale bars: 5 μm.

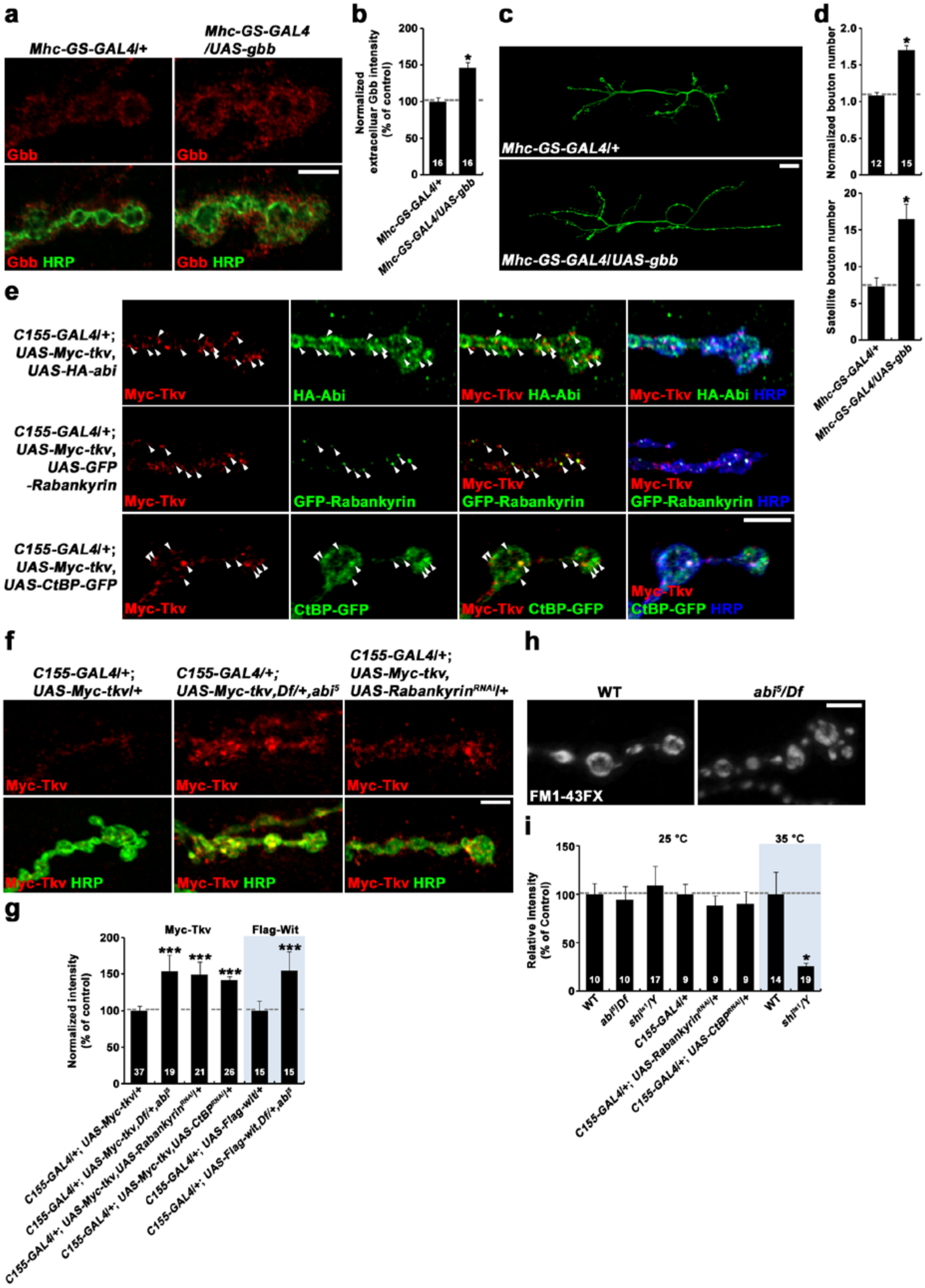


**Supplementary Figure 8.** Characterization of *Mhc-GS-GAL4/UAS-gbb* larvae, the

subcellular localization of overexpressed Myc-Tkv in NMJ boutons, and the effects of macropinocytosis on the level of surface Myc-Tkv and synaptic vesicle recycling. **a**-**d** *Mhc-GS-GAL4*/+ and *Mhc-GS-GAL4*/*UAS-gbb* larvae at third instar stage were bathed in a solution containing 3 mg/ml RU486 for 2 min before they were further allowed to develop for 8 h to wandering third instar stage on normal food. **a**, **b** GeneSwich-mediated conditional enhancement of extracellular Gbb at the NMJ 6/7 of *Mhc-GS-GAL4*/*UAS-gbb* larvae. **a** Single confocal sections of NMJ 6/7 stained with anti-Gbb and anti-HRP antibodies under nonpermeant conditions. **b** Quantification of mean extracellular Gbb to HRP fluorescence intensities. **c**, **d** *Mhc-GS-GAL4*/*UAS-gbb* larvae exposed to RU486 display synaptic overgrowth. **c** Confocal images of anti-HRP-labeled NMJ 6/7. **d** Quantification of synaptic structure. **e** Myc-Tkv partially colocalizes with HA-Abi, GFP-Rabankyrin, and CtBP-GFP in NMJ boutons. Single confocal sections of NMJ 6/7 branches from third instar larvae of indicated genotypes triply stained with anti-HRP (blue), anti-Myc (red), and anti-HA (green) antibodies. Arrowheads indicate Myc-Tkv puncta colocalizing with HA-Abi, GFP-Rabankyrin, or CtBP-GFP. **f, g** Loss of Abi or presynaptic knockdown of Rabankyrin increases levels of surface BMPRs. **f** Single confocal sections of NMJ 6/7 boutons of indicated genotypes stained with anti-HRP (green) and anti-Myc (red) under nonpermeant conditions. **g** Quantification of the ratio of surface Tkv/Wit to HRP fluorescence intensities. **h**, **i** *abi* null larvae or larvae presynaptically expressing $Rabankyrin^{RNAi}$ or $CtBP^{RNAi}$ show normal FM1-43FX dye uptake during nerve stimulation. **h** Confocal images of NMJ synapses labeled for 2 min in high-$K^+$ solution. **i** Quantification of FM1-43FX labeling intensity in indicated genotypes. Bar graphs indicate mean ± s.e.m. The number of NMJ branches (**b**, **g**, **i**) and NMJ (**d**) analyzed is indicated inside bars. Statistical analyses were performed by Student's *t*-test for **b**, **d**, **g** (Flag-Wit), and **i** (35 $^{o}$C) or by one-way ANOVA with Tukey-Kramer post hoc test

for **g** (Myc-Tkv) and **i** (25 $^{o}$C). *$P < 0.001$; ***$P < 0.05$. Scale bars: **a**, **e**, **f**, **h**, 5 μm; **c**, 20 μm.

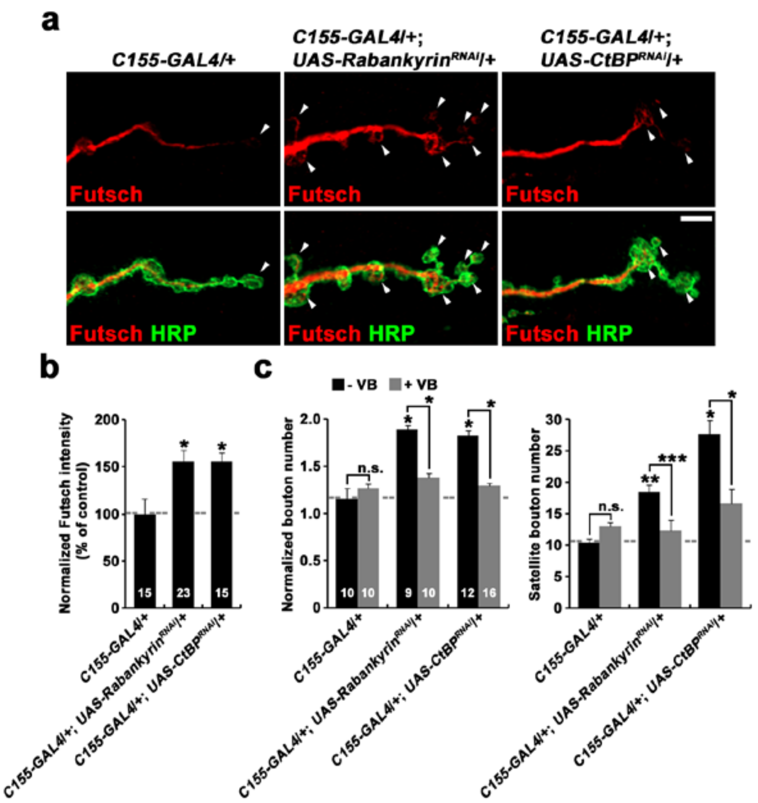


**Supplementary Figure 9.** Genetic interactions of *abi* and *Rabankyrin* or *CtBP*. **a** Presynaptic knockdown of Rabankyrin or CtBP increases Futsch/MAP1B expression. Single confocal slices of NMJ 6/7 labeled with anti-Futsch and anti-HRP in indicated genotypes. Arrowheads indicate Futsch-positive loops. **b** Quantification of Futsch normalized to HRP intensity in indicated genotypes. **c** Quantification of NMJ 6/7 bouton number in indicated genotypes reared either in the absence (- VB) or presence (+ VB) of vinblastine (1 μM). Bar graphs indicate mean ± s.e.m. NMJ number analyzed for each genotype is indicated inside bars. All comparisons against *C155-GAL4*/+ unless indicated (one-way ANOVA with Tukey-Kramer post hoc test; *$P < 0.001$; **$P < 0.01$; ***$P < 0.05$; n.s., not significant). Scale bar: 5 μm.

## Fig. 1b

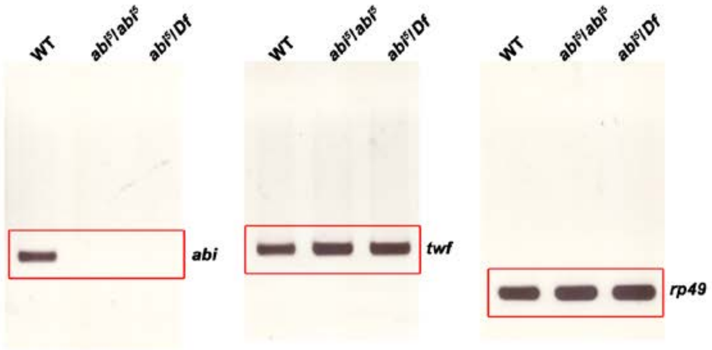


## Fig. 1e

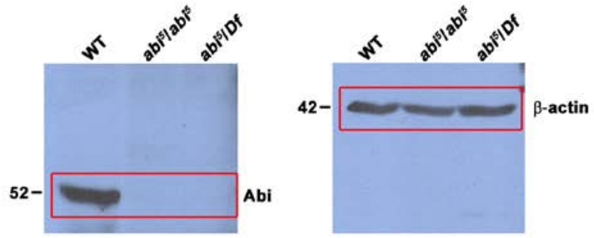


## Fig. 6e

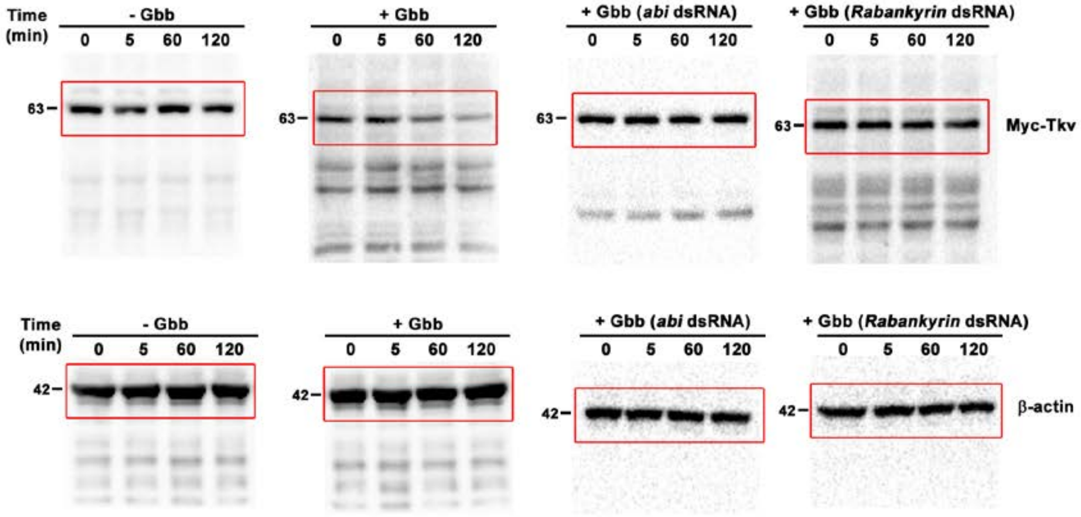

## Supplementary Figure 2b

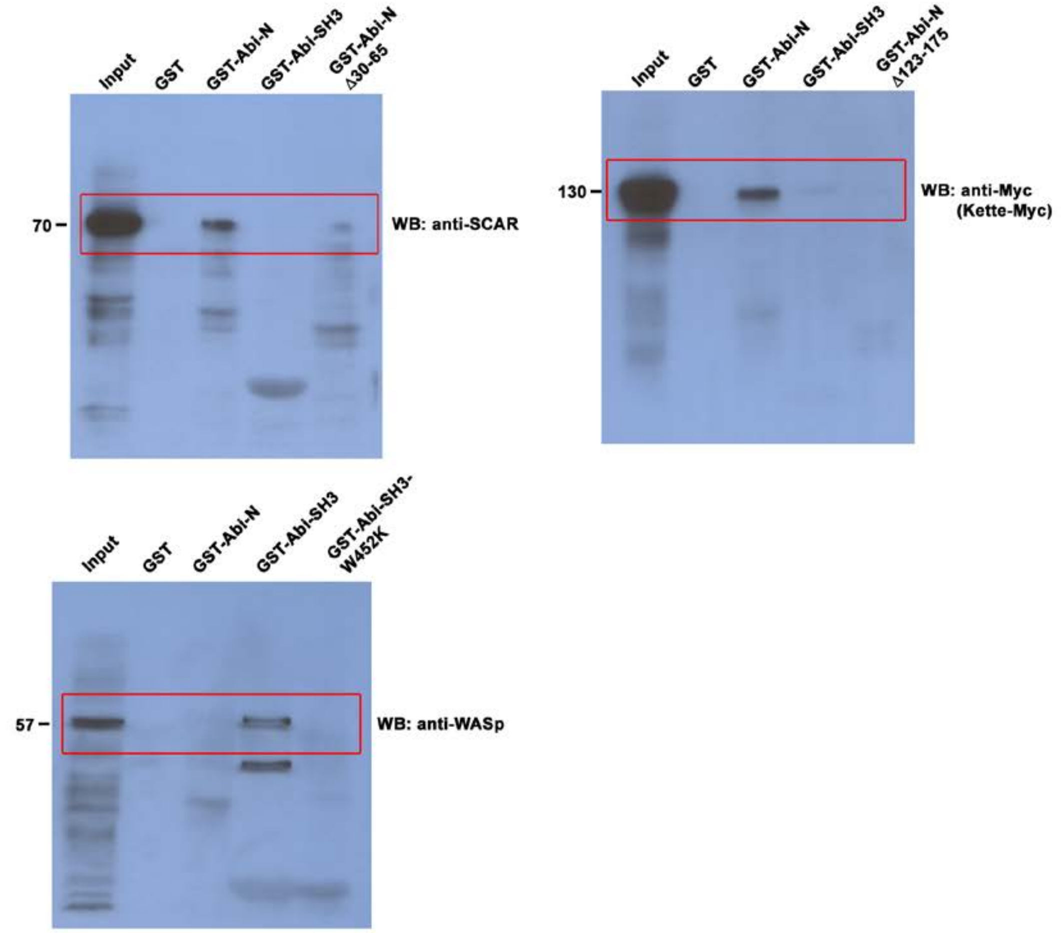


## Supplementary Figure 2c

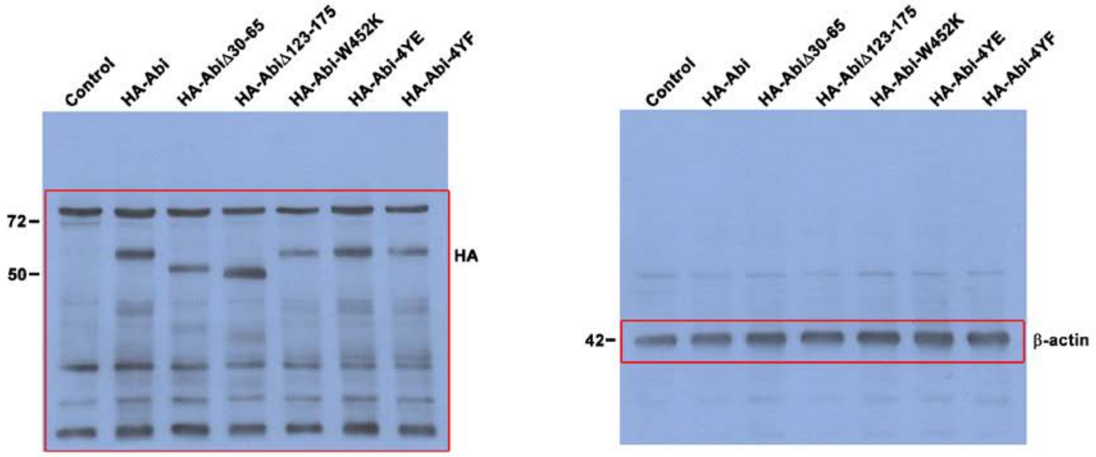

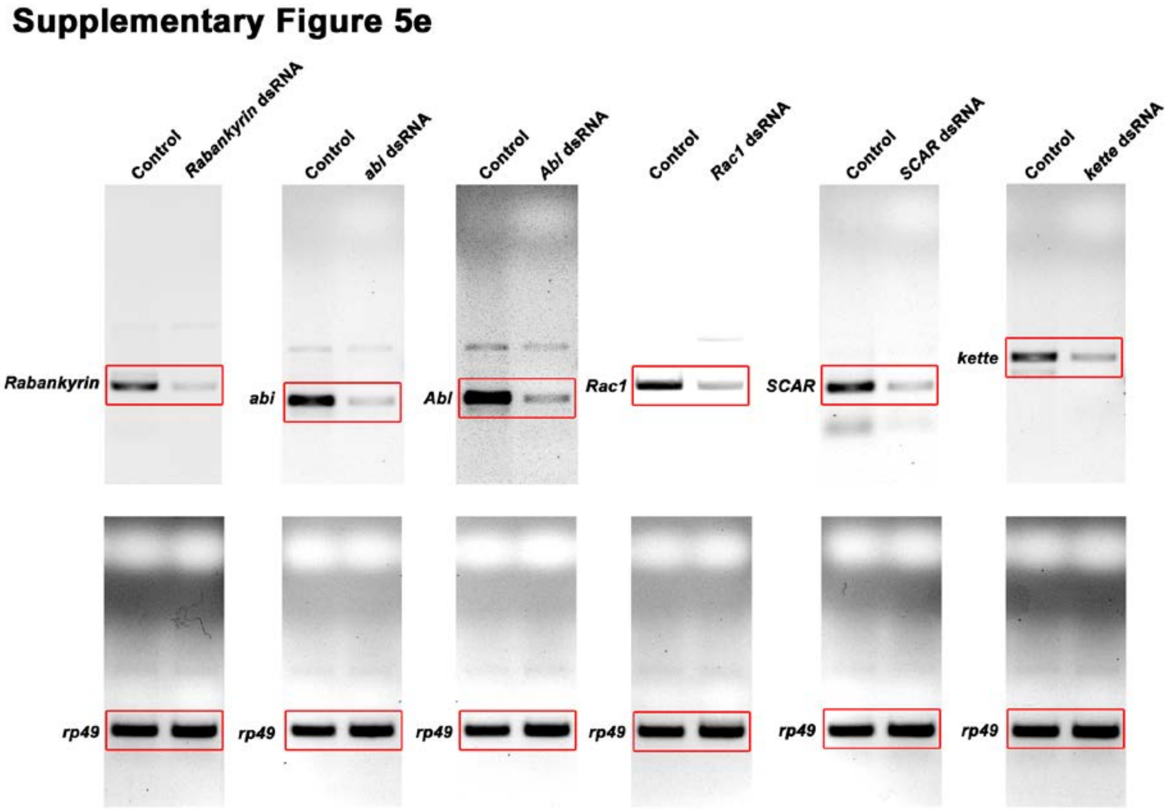


**Supplementary Figure 10.** Uncropped gels and blots. Unprocessed gel and blot images shown in Fig. 1b, 1e, 6e, and Supplementary Figure 2b, 2c, 5e. Cropped areas are indicated by red line boxes.

**Supplementary Table 1.** *Drosophila* stocks and cell lines.

| ***Drosophila*** | **Vendor** | **Stock number** |
|---|---|---|
| *Df(3R)su(Hw)7* | Bloomington Stock Center | 1049 |
| $Abl^{1}$ | Bloomington Stock Center | 3554 |
| $Abl^{4}$ | Bloomington Stock Center | 3553 |
| $Rac1^{J11}$ | Bloomington Stock Center | 6674 |
| $WASp^{1}$ | Bloomington Stock Center | 51657 |
| $wit^{A12}$ | Bloomington Stock Center | 5173 |
| $wit^{B11}$ | Bloomington Stock Center | 5174 |
| $dFmr1^{\Delta 50M}$ | Bloomington Stock Center | 6930 |
| $kette^{J4\text{-}48}$ | Bloomington Stock Center | 8753 |
| $SCAR^{\Delta 37}$ | Bloomington Stock Center | 8754 |
| $shi^{ts1}$ | Bloomington Stock Center | 7068 |
| *UAS-Abl* | Bloomington Stock Center | 28993 |
| $UAS\text{-}Abl^{K417N}$ | Bloomington Stock Center | 8566 |
| *UAS-dFmr1* | Bloomington Stock Center | 6931 |
| $UAS\text{-}Rac1^{T17N}$ | Bloomington Stock Center | 6292 |
| $UAS\text{-}Rac1^{G12V}$ | Bloomington Stock Center | 6291 |
| $UAS\text{-}SCAR^{RNAi}$ | Bloomington Stock Center | 31126 |
| $UAS\text{-}kette^{RNAi}$ | Bloomington Stock Center | 29406 |
| $UAS\text{-}Rabankyrin^{RNAi}$ | Bloomington Stock Center | 34883 |
| $UAS\text{-}CtBP^{RNAi}$ | Bloomington Stock Center | 32889 |
| **Cell line** | **Vendor** | **Stock number** |
| BG2-c2 | DGRC | 53 |
| S2R+ | DGRC | 150 |

**Supplementary Table 2.** Primers for cloning and molecular analyses.

| DNA construct | Primer sequence |
|---|---|
| *pUAST-HA-abi* | Forward: 5’-GAATTCATGTTGACCGAAACCC-3’ |
| | Reverse: 5’-GCGGCCGCTTAGACACAAGGCTCTAC-3’ |
| *pUAST-HA-abi*$^{\Delta 30-65}$ | Forward: 5’-GAATTCATGTTGACCGAAACCC-3’ |
| | Reverse: 5’-GCGGCCGCTTAGACACAAGGCTCTAC-3’ |
| | Deletion reverse: 5’-AGATCTACCACCCAGTCGCTGGCC-3' |
| | Deletion forward: 5’-AGATCTCTGGCGGCCATCGGGGAT-3' |
| *pUAST-HA-abi*$^{\Delta 123-175}$ | Forward: 5’-GAATTCATGTTGACCGAAACCC-3’ |
| | Reverse: 5’-GCGGCCGCTTAGACACAAGGCTCTAC-3’ |
| | Deletion reverse: 5’-AGATCTGCCAGGAGAGAGATTGGC-3' |
| | Deletion forward: 5’-AGATCTAAACATCGGGGCTCCAGC-3' |
| *pUAST-HA-abi*$^{4YE}$ | Forward: 5’-GAATTCATGTTGACCGAAACCC-3’ |
| | Reverse: 5’-GCGGCCGCTTAGACACAAGGCTCTAC-3’ |
| | Point mutation (Y148E+Y155E) reverse: 5’-GATTCGTCGATGGGCTTGCGCACTTCCT-3' |
| | Point mutation (Y148E+Y155E) forward: 5’-AGGAAGTGCGCAAGCCCATCGACGAATC-3' |
| | Point mutation (Y248E) reverse: 5’-GTTGGGAGCTTCGTGCGATGG-3' |
| | Point mutation (Y248E) forward: 5’-CCATCGCACGAAGCTCCCAAC-3' |
| | Point mutation (Y285E) reverse: 5’-AAGTGCACTTTCGCCAGCAGC-3' |
| | Point mutation (Y285E) forward: 5’-GCTGCTGGCGAAAGTGCACTT-3' |
| *pUAST-HA-abi*$^{4YF}$ | Forward: 5’-GAATTCATGTTGACCGAAACCC-3’ |
| | Reverse: 5’-GCGGCCGCTTAGACACAAGGCTCTAC-3’ |
| | Point mutation (Y148F+Y155F) reverse: 5’-CGAGAAGTCGATGGGCTTGCGCACAAAC-3' |
| | Point mutation (Y148F+Y155F) forward: 5’-GTTTGTGCGCAAGCCCATCGACTTCTCG-3' |
| | Point mutation (Y248F) reverse: 5’-GTTGGGAGCGAAGTGCGATGG-3' |
| | Point mutation (Y248F) forward: 5’-CCATCGCACTTCGCTCCCAAC-3' |
| | Point mutation (Y285F) reverse: 5’-GAAGTGCACTGAAGCCAGCAGC-3' |
| | Point mutation (Y285F) forward: 5’-GCTGCTGGCTTCAGTGCACTTC-3' |
| *pUAST-HA-abi*$^{W452K}$ | Forward: 5’-GAATTCATGTTGACCGAAACCC-3’ |
| | Reverse: 5’-GCGGCCGCTTAGACACAAGGCTCTAC-3’ |
| | Point mutation reverse: 5’-GCCCTCCCACTTGCCGTCGTC-3' |
| | Point mutation forward: 5’-GACGACGGCAAGTGGGAGGGC-3' |

| DNA construct | Primer sequence |
|---|---|
| *pAc-HA-abi*<br>*pAc -HA-abi*$^{\Delta 30\text{-}65}$<br>*pAc-HA-abi*$^{\Delta 123\text{-}175}$<br>*pAc-HA-abi*$^{4YE}$<br>*pAc-HA-abi*$^{4YF}$ | Forward: 5'-GAATTCGCCACCATGGCTCATAGGTACCCGTACGATGTTCCTGACTATGCGATGTTGACCGAAACCCCCATGG-3' |
| | Reverse: 5'-GCGGCCGCTTAGACACAAGGCTCTAC-3' |
| *pGEX6P1-abi-N*<br>*pGEX6P1-abi-N*$^{\Delta 30\text{-}65}$<br>*pGEX6P1-abi-N*$^{\Delta 123\text{-}175}$ | Forward: 5'-GAATTCATGTTGACCGAAACCC-3' |
| | Reverse: 5'-GCGGCCGCTTAGAAGTCTTGATGCTCATCCTC-3' |
| *pGEX6P1-abi-SH3*<br>*pGEX6P1-abi-SH3*$^{W452K}$ | Forward: 5'-GAATTCGGACGACCACGCACATCG-3' |
| | Reverse: 5'-GCGGCCGCTTAGACACAAGGCTCTAC-3' |
| *pGEX6P1-abi-C* | Forward: 5'-GAATTCCCTATTGGGCATCCAAAGCG-3' |
| | Reverse: 5'-GCGGCCGCTTAGACACAAGGCTCTAC-3' |
| *pCaSpeR4-abi promoter-GAL4* | Forward: 5'-GGATTCGGCAAACGGGAAACGAAAAC-3' |
| | Reverse: 5'-CTCGAGAAATCTGTATCTGTGTGATA-3' |
| *pAc-Myc-tkv* | Forward: 5'-GGTACCGCCACCATGTTTTGTTTTTTGTTAG-3' |
| | Reverse: 5'-CTCGAGTTAGACAATCTTAATGGGCAC-3' |
| | Myc tag reverse: 5'-CAGATCCTCTTCAGAGATGAGTTTCTGCTCACCAGTTGTTAAGTGCAC-3' |
| | Myc tag forward: 5'-GAGCAGAAACTCATCTCTGAAGAGGATCTGGAATTCAGTGCGAATG-3' |
| *pUAST-Myc-tkv* | Forward: 5'-AGATCTGCCACCATGTTTTGTTTTTTGTTAG-3' |
| | Reverse: 5'-CTCGAGTTAGACAATCTTAATGGGCAC-3' |
| *pUAST-Flag-wit* | Forward: 5'- GAATTCGCCACCATGAATTGGGCTATCTATC-3' |
| | Reverse: 5'- CTCGAGCTAGAGAATGTTGAGCAGGGAG-3' |
| | Flag tag reverse: 5'-CTTGTCATCGTCGTCCTTGTAGTCTGCTCTGCCCAGCGAG-3' |
| | Flag tag forward: 5'-GACTACAAGGACGACGATGACAAGACACCTGTTCCCAATC-3' |
| *pAc-Myc-wit* | Forward: 5'- GGTACCGCCACCATGAATTGGGCTATCTATC-3' |
| | Reverse: 5'- CTCGAGCTAGAGAATGTTGAGCAGGGAG-3' |
| | Myc tag reverse: 5'-CAGATCCTCTTCAGAGATGAGTTTCTGCTCTGCTCTGCCCAGCGAG-3' |
| | Myc tag forward: 5'-GAGCAGAAACTCATCTCTGAAGAGGATCTGACACCTGTTCCCAATC-3' |

| **DNA construct** | **Primer sequence** |
|---|---|
| *pUAST-GFP-Rabankyrin* | Forward: 5'-AGATCTATGAAAACAGGTAGTAATGAGACG-3' |
| | Reverse: 5'-GGTACCCTATGACAAGGAGCCATTTCCAC-3' |
| | GFP forward: 5'-GAATTCGCCACCATGGTGAGCAAGGGCGAGGA-3' |
| *pAc-GFP-Rabankyrin* | Forward: 5'-GAATTCATGAAAACAGGTAGTAATGAGACG-3' |
| | Reverse: 5'-CTCGAGCTATGACAAGGAGCCATTTCCAC-3' |
| *pUAST-CtBP-GFP*<br>*pAc-CtBP-GFP* | Forward: 5'-GGTACCGCCACCATGGACAAAAATCTGATGATGC-3' |
| | Reverse: 5'-GGATCCCCTTTTCTTGATTTGATATCATTTGTAGTTTACC-3' |
| *pAc-PLC-PH-GFP*<br>*pAc-PLC-PH-mCherry* | Forward: 5'-CTTGAATTCGCCACCATGGACTCGGGCCG-3' |
| | Reverse: 5'-CTCGAGCTTCAGGAAGTTCTGCAG-3' |
| *pAc-Abl-Myc*<br>*pAc-Abl*$^{K417N}$*-Myc* | Forward: 5'-GCGGCCGCGCCACCATGGGGGCTCAGCAGGGCAAG-3' |
| | Reverse: 5'-TCTAGACAGATCCTCTTCAGAGATGAGTTTCTGCTCCCTGTTAAGCGCATT GGAGATCTGACGCAG-3' |
| *pAc-GFP-Rab5* | Forward: 5'-GAATTCATGGCAACCACTCCACGC-3' |
| | Reverse: 5'-CTCGAGTCACTTGCAGCAGTTGTTCGT-3' |
| *pAc-GFP-Rab7* | Forward: 5'-GAATTCATGTCCGGACGTAAGAAA-3' |
| | Reverse: 5'-CTCGAGTTAGCACTGACAGTTGTC-3' |
| *pAc-kette-Myc* | Forward: 5'-GCGGCCGCGCCACCATGGCACGCCCAATTTTTCCGAAC-3' |
| | Reverse: 5'-TCTAGATTACAGATCCTCTTCAGAGATGAGTTTCTGCTCGAGTGCCAGCC CTAAGATCTGT-3' |
| *pAc-gbb* | Forward: 5’-GCGGCCGCGCCACCATGTCGGGACTGCGAAAC-3’ |
| | Reverse: 5’-TCTAGATCAATGGCACCCGCAGGATTTCAC-3’ |
| *pAc-HA-gbb* | Forward: 5’-GCGGCCGCGCCACCATGTCGGGACTGCGAAAC-3’ |
| | Reverse: 5’-TCTAGATCAATGGCACCCGCAGGATTTCAC-3’ |
| | HA tag reverse: 5’- TCCTCCCGCATAGTCAGGAACATCGTACGGGTATCCTCCCGTGCT CTCCATCGGTTC-3' |
| | HA tag forward: 5’- GGAGGATACCCGTACGATGTTCCTGACTATGCGGGAGGACGCAG CTGCCAGATGCAG-3’ |
| **RNA** | **Primer sequence** |
| *dFmr1 RNA* | Forward: 5'-GGATCAGAACATACCACGTG-3' |
| | Reverse: 5'-CGCCTCCACGATAGCTGCCAG-3' |

**Supplementary Table 3.** Commercial antibodies used for immunostaining and Western blotting.

| Primary antibody | Vendor | Catalog number |
|---|---|---|
| Rabbit anti-P-Smad3 | Epitomics | 1880-1 |
| Rabbit anti-HA | Cell Signaling Technology | 3724S |
| Mouse anti-HA | BioLegend | 901501 |
| Rabbit anti-Myc | Cell Signaling Technology | 2278S |
| Mouse anti-Myc | BD Pharmingen | 51-1485GR |
| Mouse anti-Flag | Sigma-Aldrich | F1804 |
| Rabbit anti-β-actin | Sigma-Aldrich | A2066 |
| Mouse anti-Dlg (4F3) | Developmental Studies Hybridoma Bank | AB_528203 |
| Mouse anti-Bruchpilot (nc82) | Developmental Studies Hybridoma Bank | AB_2314866 |
| Mouse anti-Futsch (22C10) | Developmental Studies Hybridoma Bank | AB_528403 |
| Mouse anti-Even Skipped (2B8) | Developmental Studies Hybridoma Bank | AB_528230 |
| Mouse anti-Gbb (GBB 3D6-24) | Developmental Studies Hybridoma Bank | AB_2618111 |
| Mouse anti-SCAR (P1C1-SCAR) | Developmental Studies Hybridoma Bank | AB_2618386 |
| Mouse anti-WASp (P5E1-Wasp) | Developmental Studies Hybridoma Bank | AB_2618392 |
| **Secondary antibody** | **Vendor** | **Catalog number** |
| FITC-conjugated anti-mouse secondary antibody | Jackson ImmunoResearch Laboratories | 715-095-150 |
| | | 715-095-151 |
| FITC-conjugated anti-rabbit secondary antibody | Jackson ImmunoResearch Laboratories | 711-095-152 |
| Cy3-conjugated anti-mouse secondary antibody | Jackson ImmunoResearch Laboratories | 715-165-150 |
| Cy3-conjugated anti-rabbit secondary antibody | Jackson ImmunoResearch Laboratories | 711-165-152 |
| Cy3-conjugated anti-rat secondary antibody | Jackson ImmunoResearch Laboratories | 712-165-153 |
| Cy5-conjugated anti-rabbit secondary antibody | Jackson ImmunoResearch Laboratories | 711-175-152 |
| Cy5-conjugated anti-rat secondary antibody | Jackson ImmunoResearch Laboratories | 712-175-153 |
| FITC-conjugated goat anti-HRP | Jackson ImmunoResearch Laboratories | 123-095-021 |
| Cy5-conjugated goat anti-HRP | Jackson ImmunoResearch Laboratories | 123-175-021 |
| Peroxidase-conjugated anti-rabbit secondary antibody | Jackson ImmunoResearch Laboratories | 111-035-144 |
| Peroxidase-conjugated anti-rat secondary antibody | Jackson ImmunoResearch Laboratories | 112-035-062 |

**Supplementary References**


1. Martinez-Quiles, N., Ho, H.Y., Kirschner, M.W., Ramesh, N. & Geha, R.S. Erk/Src phosphorylation of cortactin acts as a switch on-switch off mechanism that controls its ability to activate N-WASP. *Mol.Cell. Biol.* **24**, 5269-5280 (2004).
2. Buckley, C.M. & King, J.S. Drinking problems: mechanisms of macropinosome formation and maturation. *FEBS Lett.* **284**, 3778-3790 (2017).
3. Liberali, P., *et al.* The closure of Pak1-dependent macropinosomes requires the phosphorylation of CtBP1/BARS. *EMBO J.* **27**, 970-981 (2008).
