# Supplementary material for: BMP-dependent synaptic development requires Abi-Abl-Rac signaling of BMP receptor macropinocytosis
Source: Nat Commun. 2019 Feb 8;10:684. doi: 10.1038/s41467-019-08533-2 (PMC6368546; doi:10.1038/s41467-019-08533-2)
Supplement: Supplementary file 2 — Reporting Summary [file 41467_2019_8533_MOESM2_ESM.pdf]

nature research

Corresponding author(s): Seungbok Lee

Last updated by author(s): Dec 26, 2018

Reporting Summary

Nature Research wishes to improve the reproducibility of the work that we publish. This form provides structure for consistency and transparency in reporting. For further information on Nature Research policies, see Authors & Referees and the Editorial Policy Checklist.

Statistics

For all statistical analyses, confirm that the following items are present in the figure legend, table legend, main text, or Methods section.

n/a	Confirmed	
☐	☒	The exact sample size (*n*) for each experimental group/condition, given as a discrete number and unit of measurement
☐	☒	A statement on whether measurements were taken from distinct samples or whether the same sample was measured repeatedly
☐	☒	The statistical test(s) used AND whether they are one- or two-sided<br>*Only common tests should be described solely by name; describe more complex techniques in the Methods section.*
☒	☐	A description of all covariates tested
☐	☒	A description of any assumptions or corrections, such as tests of normality and adjustment for multiple comparisons
☐	☒	A full description of the statistical parameters including central tendency (e.g. means) or other basic estimates (e.g. regression coefficient) AND variation (e.g. standard deviation) or associated estimates of uncertainty (e.g. confidence intervals)
☐	☒	For null hypothesis testing, the test statistic (e.g. *F*, *t*, *r*) with confidence intervals, effect sizes, degrees of freedom and *P* value noted<br>*Give P values as exact values whenever suitable.*
☒	☐	For Bayesian analysis, information on the choice of priors and Markov chain Monte Carlo settings
☒	☐	For hierarchical and complex designs, identification of the appropriate level for tests and full reporting of outcomes
☒	☐	Estimates of effect sizes (e.g. Cohen's *d*, Pearson's *r*), indicating how they were calculated

Our web collection on statistics for biologists contains articles on many of the points above.

Software and code

Policy information about availability of computer code

Data collection	Imaging data of Drosophila larval neuromuscular junctions (NMJs), ventral nerve cords, and BG2-c2 cells were collected on confocal microscopes using ZEN 2.3 (blue edition, Carl Zeiss) and OLYMPUS FLUOVIEW Ver.5.0 (Olympus) softwares. Time-lapse imaging of BG2-c2 cells was performed ZEN 2.3 software (blue edition, Carl Zeiss).For chemiluminescent Western blotting, X-ray film exposure (Fig. 1e; Supplementary Fig. 2b, c) or ChemiDoc MP Imaging System with Image Lab Software (BIO-RAD) (Fig. 6e) were used. Quantitative real-time PCR data were obtained using 7500 Software for 7500 and 7500 Fast Real Time PCR Systems v2.0.6 (Applied Biosystems by Life technologies).
Data analysis	NMJ bouton number and muscle surface area were analyzed using ImageJ Version 1.5h (https://fiji.sc/#download). The fluorescence intensities of larval tissues and BG2-c2 cells were analyzed using ZEN 2.3 software (blue edition, Carl Zeiss). Quantitative analysis of macropinosome number and co-localization between TMR-Dex, Abi/endosomal makers, and BMP receptors was performed using ImageJ Version 1.5h software (https://fiji.sc/#download). Statistical analysis of data (one-way ANOVA with post hoc Tukey–Kramer test or student's t-test) was performed using SigmaPlot 11.0 (Systat Software, Inc.).

For manuscripts utilizing custom algorithms or software that are central to the research but not yet described in published literature, software must be made available to editors/reviewers. We strongly encourage code deposition in a community repository (e.g. GitHub). See the Nature Research guidelines for submitting code & software for further information.

Data

Policy information about availability of data

All manuscripts must include a data availability statement. This statement should provide the following information, where applicable:

- Accession codes, unique identifiers, or web links for publicly available datasets
- A list of figures that have associated raw data
- A description of any restrictions on data availability

All data supporting the findings of this study are available within the article and its Supplementary Information files. All other data supporting the findings of this

study are available from the corresponding author upon reasonable request.

Field-specific reporting

Please select the one below that is the best fit for your research. If you are not sure, read the appropriate sections before making your selection.

☒ Life sciences ☐ Behavioural & social sciences ☐ Ecological, evolutionary & environmental sciences

For a reference copy of the document with all sections, see nature.com/documents/nr-reporting-summary-flat.pdf

Life sciences study design

All studies must disclose on these points even when the disclosure is negative.

Sample size	Typical sample sizes where chosen in accordance with previous publications and are similar to those generally employed in the field. All the experiments were repeated at least three times independently.
Data exclusions	No data were excluded from the analysis.
Replication	All experimental finding were reliably reproducible.
Randomization	No randomization was applied. However, for immunostaining, all genotypes were prepared in one session, stained in one tube and strictly analyzed in an unbiased manner.
Blinding	Investigators were not blinded to Drosophila genotypes during experiments.

Reporting for specific materials, systems and methods

We require information from authors about some types of materials, experimental systems and methods used in many studies. Here, indicate whether each material, system or method listed is relevant to your study. If you are not sure if a list item applies to your research, read the appropriate section before selecting a response.

Materials & experimental systems

n/a	Involved in the study	
☐	☒	Antibodies
☐	☒	Eukaryotic cell lines
☒	☐	Palaeontology
☐	☒	Animals and other organisms
☒	☐	Human research participants
☒	☐	Clinical data

Methods

n/a	Involved in the study	
☒	☐	ChIP-seq
☒	☐	Flow cytometry
☒	☐	MRI-based neuroimaging

Antibodies

Antibodies used

Antibodies used for immunostaining and western blot: Species, Antibody name (supplier name, catalog number, clone name)

Rat anti-Abi (Seungbok Lee, Republic of Korea, see Fig.1e, f)
rabbit anti-Gbb (Seungbok Lee, Republic of Korea, PLoS genetics 8, e1003031 (2012))
rabbit anti-pMad (FEBS Lett 434, 83-87 (1998), PS1)
rabbit anti-GluRIIC (Journal of Neuroscience 24 (6), 1406-1415 (2004))
rabbit anti-HA (Cell Signaling Technology, 3724S, C29F4)
rabbit anti-Myc (Cell Signaling Technology, 2278S, 71D10)
mouse anti-Flag (Sigma-Aldrich, F1804, M2)
mouse anti-Futsch (Developmental Studies Hybridoma Bank, 22C10)
mouse anti-Bruchpilot (Developmental Studies Hybridoma Bank, nc82)
mouse anti-Dlg (Developmental Studies Hybridoma Bank, 4F3)
rat anti-Even Skipped (Developmental Studies Hybridoma Bank, 2B8)
rabbit anti-pSmad3 (Epitomics, 1880-1, EP823Y)
mouse anti-HA (BioLegend, 901501, 16B12)
mouse anti-Myc antibody (BD pharmingen, 551101, 9E10)
rabbit anti-beta-actin (Sigma-Aldrich, A2066)
mouse anti-SCAR (Developmental Studies Hybridoma Bank, P1C1-SCAR)
mouse anti-WASp (Developmental Studies Hybridoma Bank, P5E1-Wasp)
mouse anti-Gbb (Developmental Studies Hybridoma Bank, GBB 3D6-24)
Fluorescein (FITC) AffiniPure Goat Anti-Horseradish Peroxidase, (Jackson ImmunoResearch Laboratories, 123-095-021)
Cy™5 AffiniPure Goat Anti-Horseradish Peroxidase, (Jackson ImmunoResearch Laboratories, 123-175-021)
Fluorescein (FITC) AffiniPure Donkey Anti-Mouse IgG (H+L), (Jackson ImmunoResearch Laboratories, 715-095-151)

	Fluorescein (FITC) AffiniPure Donkey Anti-Mouse IgG (H+L), (Jackson ImmunoResearch Laboratories, 715-095-150)<br>Cy™3 AffiniPure Donkey Anti-Mouse IgG (H+L), (Jackson ImmunoResearch Laboratories, 715-165-150)<br>Fluorescein (FITC) AffiniPure Donkey Anti-Rat IgG (H+L), (Jackson ImmunoResearch Laboratories, 712-095-153)<br>Cy™3 AffiniPure Donkey Anti-Rat IgG (H+L), (Jackson ImmunoResearch Laboratories, 712-165-153)<br>Cy™5 AffiniPure Donkey Anti-Rat IgG (H+L), (Jackson ImmunoResearch Laboratories, 712-175-153)<br>Fluorescein (FITC) AffiniPure Donkey Anti-Rabbit IgG (H+L), (Jackson ImmunoResearch Laboratories, 711-095-152)<br>Cy™3 AffiniPure Donkey Anti-Rabbit IgG (H+L), (Jackson ImmunoResearch Laboratories, 711-165-152)<br>Cy™5 AffiniPure Donkey Anti-Rabbit IgG (H+L), (Jackson ImmunoResearch Laboratories, 711-175-152)<br>Peroxidase AffiniPure Donkey Anti-Mouse IgG (H+L), (Jackson ImmunoResearch Laboratories, 715-035-150)<br>Peroxidase AffiniPure Goat Anti-Rat IgG (H+L), (Jackson ImmunoResearch Laboratories, 112-035-062)<br>Peroxidase AffiniPure Goat Anti-Rabbit IgG (H+L), (Jackson ImmunoResearch Laboratories, 111-035-144)
Validation	rabbit anti-HA (Cell Signaling Technology, 3724S, C29F4) https://www.cellsignal.com/products/primary-antibodies/ha-tag-c29f4-rabbit-mab/3724<br>rabbit anti-Myc (Cell Signaling Technology, 2278S, 71D10) https://www.cellsignal.com/products/primary-antibodies/myc-tag-71d10-rabbit-mab/2278<br>mouse anti-Flag (Sigma-Aldrich, F1804, M2) https://www.sigmaaldrich.com/catalog/product/sigma/f1804?lang=ko&region=KR<br>mouse anti-Futsch (Developmental Studies Hybridoma Bank, 22C10, Monoclonal) http://dshb.biology.uiowa.edu/futsch<br>mouse anti-Bruchpilot (Developmental Studies Hybridoma Bank, nc82) http://dshb.biology.uiowa.edu/bruchpilot<br>mouse anti-Dlg (Developmental Studies Hybridoma Bank, 4F3) http://dshb.biology.uiowa.edu/4F3-anti-discs-large<br>rat anti-Even Skipped (Developmental Studies Hybridoma Bank, 2B8) http://dshb.biology.uiowa.edu/2B8<br>rabbit anti-pSmad3 (Epitomics, 1880-1, EP823Y) https://www.labome.com/product/Epitomics/1880-1.html<br>mouse anti-HA.11 (BioLegend, 901501, 16B12) https://www.biolegend.com/en-us/products/purified-anti-ha-11-epitope-tag-antibody-11374<br>mouse anti-Myc antibody (BD bioscience, 551101, 9E10) http://www.bdbiosciences.com/us/applications/research/apoptosis/purified-antibodies/purified-mouse-anti-human-c-myc-with-control/p/551101<br>rabbit anti-beta-actin (Sigma-Aldrich, A2066) https://www.sigmaaldrich.com/catalog/product/sigma/a2066?lang=ko&region=KR<br>mouse anti-SCAR (Developmental Studies Hybridoma Bank, P1C1-SCAR) http://dshb.biology.uiowa.edu/SCAR<br>mouse anti-WASp (Developmental Studies Hybridoma Bank, P5E1-Wasp) http://dshb.biology.uiowa.edu/P5E1-Wasp<br>mouse anti-Gbb (Developmental Studies Hybridoma Bank, GBB 3D6-24) http://dshb.biology.uiowa.edu/glass-bottom-boat

Eukaryotic cell lines

Policy information about cell lines

Cell line source(s)	S2R+ cells were obtained from Drosophila Genomics Resource Center (stock#150)<br>BG2-c2 (ML-DmBG2-c2) were obtained from Drosophila Genomics Resource Center (stock#53)
Authentication	None of the cell lines were authenticated.
Mycoplasma contamination	The cell lines were not tested for mycoplasma contamination.
Commonly misidentified lines<br>(See ICLAC register)	Commonly misidentified cell lines were not used.

Animals and other organisms

Policy information about studies involving animals; ARRIVE guidelines recommended for reporting animal research

Laboratory animals	For all experiments, Drosophila melanogaster (fruit flies) were used.
Wild animals	The study did not involve wild animals.
Field-collected samples	The study did not involve samples collected from the field.
Ethics oversight	NA

Note that full information on the approval of the study protocol must also be provided in the manuscript.
